# Consumption of cephalosporins in the community, European Union/European Economic Area, 1997–2017

**DOI:** 10.1093/jac/dkab174

**Published:** 2021-08-01

**Authors:** Ann Versporten, Robin Bruyndonckx, Niels Adriaenssens, Niel Hens, Dominique L Monnet, Geert Molenberghs, Herman Goossens, Klaus Weist, Samuel Coenen, the ESAC-Net study group, Reinhild Strauss, Eline Vandael, Stefana Sabtcheva, Marina Payerl-Pal, Isavella Kyriakidou, Jiří Vlček, Ute Wolff Sönksen, Elviira Linask, Emmi Sarvikivi, Karima Hider-Mlynarz, Katja Niepraschk-von Dollen, Flora Kontopidou, Mária Matuz, Gudrun Aspelund, Karen Burns, Filomena Fortinguerra, Elīna Dimiņa, Jolanta Kuklytė, Marcel Bruch, Peter Zarb, Stephanie Natsch, Hege Salvesen Blix, Anna Olczak-Pieńkowska, Ana Silva, Gabriel Adrian Popescu, Tomáš Tesař, Milan Čižman, Antonio López Navas, Vendela Bergfeldt, Berit Müller-Pebody

**Affiliations:** 1 Laboratory of Medical Microbiology, Vaccine & Infectious Disease Institute (VAXINFECTIO), University of Antwerp, Antwerp, Belgium; 2 Interuniversity Institute for Biostatistics and statistical Bioinformatics (I-BIOSTAT), Hasselt University, Data Science Institute, Hasselt, Belgium; 3 Centre for General Practice, Department of Family Medicine & Population Health (FAMPOP), University of Antwerp, Antwerp, Belgium; 4 Centre for Health Economic Research and Modelling Infectious Diseases, Vaccine & Infectious Disease Institute (VAXINFECTIO), University of Antwerp, Antwerp, Belgium; 5 Disease Programmes Unit, European Centre for Disease Prevention and Control, Stockholm, Sweden; 6 Interuniversity Institute for Biostatistics and statistical Bioinformatics (I-BIOSTAT), Catholic University of Leuven, Leuven, Belgium

## Abstract

**Objectives:**

Data on cephalosporin consumption in the community were collected from 30 EU/EEA countries over two decades. This article reviews temporal trends, seasonal variation, presence of change-points and changes in the composition of the main subgroups of cephalosporins.

**Methods:**

For the period 1997–2017, data on consumption of cephalosporins (i.e. first-, second-, third- and fourth-generation cephalosporins; ATC subgroups J01DB, J01DC, J01DD and J01DE, respectively) in the community and aggregated at the level of the active substance, were collected using the WHO ATC/DDD methodology (ATC/DDD index 2019). Consumption was expressed in DDD per 1000 inhabitants per day and in packages per 1000 inhabitants per day. Cephalosporin consumption was analysed based on ATC-4 subgroup, and presented as trends, seasonal variation, presence of change-points and compositional changes.

**Results:**

In 2017, cephalosporin consumption in the community expressed in DDD per 1000 inhabitants per day varied by a factor of 285 between countries with the highest (Greece) and the lowest (the Netherlands) consumption. Cephalosporin consumption did not change significantly between the first quarter of 1997 and the last quarter of 2017. Seasonal variation decreased significantly over time. Proportional consumption of second- and third-generation cephalosporins significantly increased over time compared with that of first-generation cephalosporins, and proportional consumption of fourth-generation cephalosporins significantly decreased compared with that of second- and third-generation cephalosporins.

**Conclusions:**

Despite considerable variation between countries in the composition of cephalosporin consumption and trends over time, a significant shift towards consumption of more broad-spectrum cephalosporins in the community was observed across the EU/EEA during 1997–2017.

## Introduction

This article presents data from the European Surveillance of Antimicrobial Consumption Network (ESAC-Net,^1^ formerly ESAC) on consumption of cephalosporins in the community (i.e. primary care sector) for 30 EU/EEA countries in 2017. Because consumption of monobactams, carbapenems and other cephalosporins and penems is very small in the community in EU/EEA countries, this article focusses on consumption of first-, second-, third- and fourth-generation cephalosporins (Table 1).^2^ The present study updates previous ESAC studies published in 2006 and 2011, and in doing so it provides updated comparable and reliable information on antibiotic consumption that can aid in fighting the global problem of antimicrobial resistance.^3^^,^^4^ In 2017, cephalosporins represented 11.6% of antibiotic consumption in the community.^5^ The objective of this study was to analyse temporal trends, seasonal variation and the presence of change-points in cephalosporin consumption in the community for the period 1997–2017, as well as to analyse the composition of cephalosporin consumption over time.

**Table 1.** Classification of cephalosporins (J01DB, J01DC, J01DD and J01DE; ATC/DDD index 2019)

First-generation		Second-generation		Third-generation		Fourth-generation	
J01DB01	**Cefalexin** ^a^	J01DC01	Cefoxitin	J01DD01	Cefotaxime	J01DE01	Cefepime
J01DB02	*Cefaloridine* ^b^	J01DC02	**Cefuroxime** ^a^	J01DD02	Ceftazidime	J01DE02	*Cefpirome*
J01DB03	Cefalotin	J01DC03	*Cefamandole*	J01DD03	*Cefsulodin* ^b^	J01DE03	*Cefozopran* ^b^
J01DB04	Cefazolin	J01DC04	**Cefaclor** ^a^	J01DD04	Ceftriaxone		
J01DB05	Cefadroxil	J01DC05	*Cefotetan* ^b^	J01DD05	*Cefmenoxime* ^b^		
J01DB06	*Cefazedone* ^b^	J01DC06	Cefonicide	J01DD06	*Latamoxef* ^b^		
J01DB07	Cefatrizine	J01DC07	Cefotiam	J01DD07	*Ceftizoxime*		
J01DB08	*Cefapirin* ^b^	J01DC08	*Loracarbef*	J01DD08	**Cefixime** ^a^		
J01DB09	Cefradine	J01DC09	*Cefmetazole* ^b^	J01DD09	Cefodizime		
J01DB10	*Cefacetrile* ^b^	J01DC10	Cefprozil^a^	J01DD10	*Cefetamet* ^b^		
J01DB11	*Cefroxadine* ^b^	J01DC11	Ceforanide	J01DD11	*Cefpiramide* ^b^		
J01DB12	*Ceftezole* ^b^	J01DC12	*Cefminox* ^c^	J01DD12	Cefoperazone		
		J01DC13	*Cefbuperazone* ^c^	J01DD13	**Cefpodoxime** ^a^		
		J01DC14	*Flomoxef* ^c^	J01DD14	Ceftibuten		
				J01DD15	*Cefdinir* ^b^		
				J01DD16	Cefditoren		
				J01DD17	*Cefcapene* ^b^		
				J01DD51	*Cefotaxime* and *BLI*^c^		
				J01DD52	*Ceftazidime* and *BLI*^c^		
				J01DD54	*Ceftriaxone* *combinations*		
				J01DD62	Cefoperazone and BLI		
				J01DD63	*Ceftriaxone* and *BLI*^c^		

BLI, β-lactamase inhibitor; **Bold type** indicates that consumption was part of the top 90% of the community consumption of cephalosporins (J01DB, J01DC, J01DD and J01DE) in 28 EU/EEA countries in 2017; *Italic type* indicates that no consumption of this cephalosporin was reported in 28 EU/EEA countries in 2017.

a Consumption was part of the top 90% of the community consumption of cephalosporins (J01DB, J01DC, J01DD and J01DE) in 30 EU/EEA countries in 2009.

b No consumption of this cephalosporin was reported in 30 EU/EEA countries in 2009.

c This cephalosporin was not included in the ATC/DDD index in 2009 and was therefore not reported in 2009.

## Methods

The methods for collecting and analysing the data are described in the introductory article of this series.^6^ In summary, data on consumption of cephalosporins, i.e. first-, second-, third- and fourth-generation cephalosporins (ATC groups J01DB, J01DC, J01DD and J01DE, respectively), aggregated at the level of the active substance, were collected using the WHO ATC/DDD methodology (ATC/DDD index 2019) and expressed in DDD per 1000 inhabitants per day. In addition, where data were available, cephalosporin consumption was expressed in packages per 1000 inhabitants per day. There are 51 unique ATC codes for cephalosporins in the ATC/DDD index 2019. Compared with previous descriptions of the consumption of cephalosporins in the community,^4^ three additional second-generation cephalosporins and three additional third-generation cephalosporins have been assigned an ATC code by the WHO (Table 1).

The evolution of the number of DDD per package over time was assessed using a linear mixed model. The temporal trend, seasonal variation and presence of change-points in cephalosporin consumption were assessed using a non-linear change-point mixed model fitted to quarterly data expressed in DDD per 1000 inhabitants per day from 1997 to 2017.^7^ The relative proportions of the main subgroups were assessed through a compositional data analysis modelling yearly data expressed in DDD per 1000 inhabitants per day from 1997 to 2017.^8^

## Results

An overview of consumption of cephalosporins (ATC J01DB, J01DC, J01DD and J01DE) in the community, expressed in DDD and packages per 1000 inhabitants per day for all participating countries between 1997 and 2017 is available as Supplementary data at *JAC* Online (Tables S1 and S2, respectively).

### Cephalosporin consumption in the community in 2017

In 2017, five substances accounted for 90% of cephalosporin consumption in the community expressed in DDD per 1000 inhabitants per day: cefuroxime (63.0% in 2017 compared with 56.5% in 2009), cefixime (8.9% in 2017 compared with 7.3% in 2009), cefalexin (8% in 2017 compared with 9.5% in 2009), cefaclor (7.2% in 2017 compared with 10.1% in 2009) and cefpodoxime (3.1% in 2017 compared with 3.1% in 2009) (Table 1).

**Figure 1.**
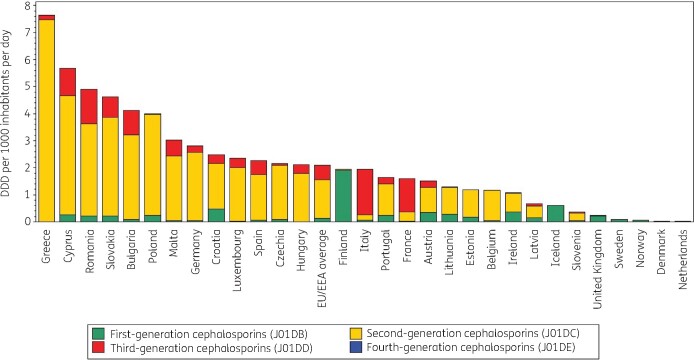
Consumption of cephalosporins (ATC J01DB, J01DC, J01DD and J01DE) in the community, expressed in DDD (ATC/DDD index 2019) per 1000 inhabitants per day, 30 EU/EEA countries, 2017. For Czechia, 2015 data are used. For Slovakia, 2016 data are used. For Cyprus and Romania, total care data, community and hospital sector combined, are used.

Figure 1 shows the consumption of cephalosporins in the community subdivided into the four generations expressed in DDD per 1000 inhabitants per day for 30 EU/EEA countries in 2017. Cephalosporin consumption in the community varied by a factor of 285 between the countries with the highest (7.65 DDD per 1000 inhabitants per day in Greece) and the lowest (0.03 DDD per 1000 inhabitants per day in the Netherlands) consumption, which was similar compared with 2009 (factor of 267, from 8.7 DDD per 1000 inhabitants per day in Greece to 0.03 DDD per 1000 inhabitants per day in Denmark). Inter-country variations were high when comparing consumption of first-, second-, third- and fourth-generation cephalosporins (Table S1).

In 2017, first-generation cephalosporins represented 10.6% (14% in 2009) of cephalosporin consumption in the community. Consumption was the highest in Northern EU/EEA countries such as Finland (98.9% of cephalosporin consumption in the community), Iceland (98.2%), Sweden (97.3%), Norway (90.3%) and the United Kingdom (89.8%) and the lowest (<1%) in Greece, Hungary and Luxembourg. First-generation cephalosporin consumption was mostly represented by one substance, namely cefalexin (J01DB01), in Croatia, Finland and Malta (100%) and Iceland (99.4%). Cefadroxil (J01DB05) was the preferred first-generation cephalosporin in Estonia and Latvia (>90%) and in Lithuania (>80%). Cefradine (J01DB09) was only consumed in the United Kingdom (5.6%), Portugal (3.8%) and France (2.2%). First-generation cephalosporins represented <5% of cephalosporin consumption in the community in Belgium, Bulgaria, France, Germany, Greece, Hungary, Italy, Luxembourg, Malta, Spain, Romania (total care data, i.e. community and hospital sector combined) and Cyprus (total care data).

In 2017, second-generation cephalosporins represented 73.0% (71.5% in 2009) of cephalosporin consumption in the community. The second-generation cephalosporins represented >60% of cephalosporin consumption in the community in 21 EU/EEA countries. Consumption was the highest for cefuroxime (J01DC02), which represented >50% of second-generation cephalosporin consumption in the community in all but three countries (Ireland, Sweden, United Kingdom). Ireland had the highest consumption of cefaclor (J01DC04; 60.4%). Countries with the highest consumption of cefprozil (J01DC10) were Hungary (16.2%), Greece (10.3%) and Estonia (6.6%). Greece was the only country using ceforanide (J01DC11).

In 2017, third-generation cephalosporins represented 16.4% (14.5% in 2009) of cephalosporin consumption in the community. These were mostly cefixime (J01DD08), cefpodoxime (J01DD13) and ceftriaxone (J01DD04), from which 20.6% was for parenteral use (mainly ceftriaxone). The highest consumption of third-generation cephalosporins was observed for Italy (>80% of cephalosporin consumption, of which mainly cefixime for oral use), France (>70%, mainly the oral substances cefpodoxime and cefixime), Romania (total care data: 26.0%, mainly cefixime and ceftriaxone), and Cyprus (total care data: 18.1%, mainly cefixime).

In 2017, 16 EU/EEA countries reported consumption of fourth-generation cephalosporins in the community. This consumption was very low, only representing 0.01% (0.02% in 2009) of cephalosporin consumption in the community in the EU/EEA. The highest consumption of fourth-generation cephalosporins was observed for Italy (0.15%). The only fourth-generation cephalosporin consumed was cefepime (J01DE01), with the exception of cefpirome (J01DE02) in Bulgaria.

**Figure 2.**
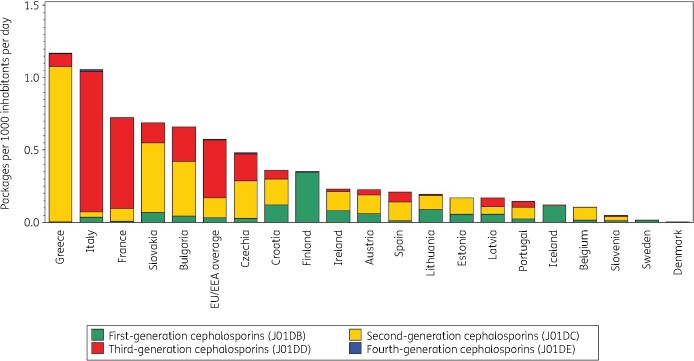
Consumption of cephalosporins (ATC J01DB, J01DC, J01DD and J01DE combined) in the community, expressed in packages per 1000 inhabitants per day, 20 EU/EEA countries, 2017. For Czechia, 2015 data are used. For Slovakia, 2016 data are used. For Cyprus and Romania, total care data, i.e. community and hospital sector combined, are used.

Figure 2 shows cephalosporin consumption in the community expressed in packages per 1000 inhabitants per day for 20 EU/EEA countries in 2017. Portugal ranked 9th for its cephalosporin consumption in DDD per 1000 inhabitants per day and 15th in packages per 1000 inhabitants per day, Spain 5th and 11th, Italy 8th and 2nd, and France 10th and 3rd, respectively (Table 2). The number of DDD per package ranged from 1.8 in Italy to 11.4 in Portugal. In the EU/EEA countries, the number of DDD per package did not change significantly over time during the period 1997–2017.

**Table 2.** Ranking of consumption of cephalosporins (ATC J01DB, J01DC, J01DD and J01DE combined) in the community, expressed in DDD or packages per 1000 inhabitants per day, 20 EU/EEA countries, 2017

Country	Greece	Italy	France	Slovakia	Bulgaria	Czechia	Croatia	Finland	Ireland	Austria	Spain	Lithuania	Estonia	Latvia	Portugal	Iceland	Belgium	Slovenia	Sweden	Denmark
Ranking for packages per 1000 inhabitants per day	1	2	3	4	5	6	7	8	9	10	11	12	13	14	15	16	17	18	19	20
Ranking for DDD per 1000 inhabitants per day	1	8	10	2	3	6	4	7	15	11	5	12	13	16	9	17	14	18	19	20
Number of DDD per package	6.5	1.8	2.2	6.7	6.2	4.5	6.9	5.5	4.6	6.7	10.9	6.7	7.0	3.9	11.4	5.0	11.2	7.8	4.6	9.9

For Czechia, 2015 data are used. For Slovakia, 2016 data are used. For Cyprus and Romania, total care data, i.e. community and hospital sector combined, are used.

### Longitudinal data analysis, 1997–2017

**Figure 3.**
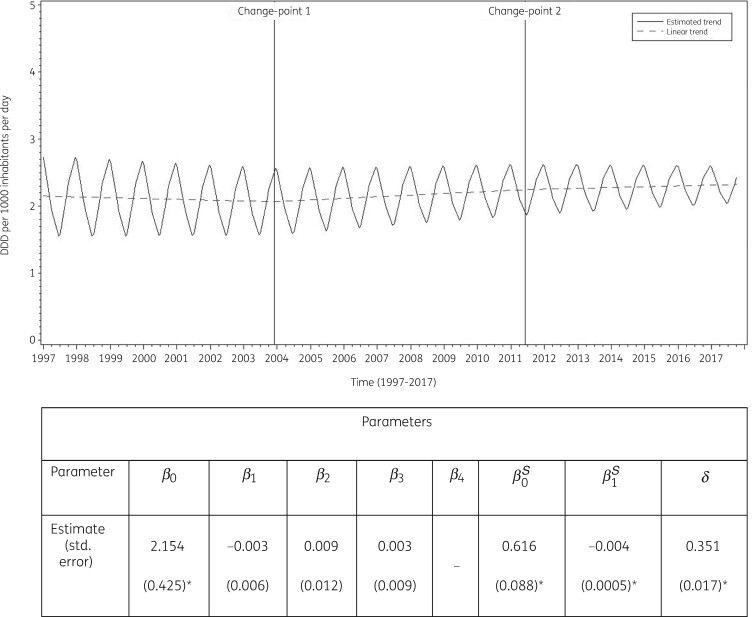
Estimated trend (solid line) and linear trend (dashed line) of consumption of cephalosporins (ATC J01DB, J01DC, J01DD and J01DE) in the community based on quarterly data, 25 EU/EEA countries, 1997–2017. *β_0_*, predicted consumption in the first quarter of 1997; *β_1_*, predicted increase (if positive)/decrease (if negative) in consumption per quarter; *β_2_*, predicted difference in slope after versus before the first change-point; *β_3_*, predicted difference in slope after versus before the second change-point; *β_4_*, predicted difference in slope after versus before the third change-point; *β_0_^S^*, predicted amplitude of the upward winter and downward summer peak in consumption; *β_1_^S^*, predicted increase (if positive)/decrease (if negative) of the amplitude of the upward winter and downward summer peak in consumption per quarter; *δ*, shift in timing of the upward winter and downward summer peak from one year to another. An asterisk indicates the result is statistically significant at significance level 0.05.

The best fit was obtained for a model including two change-points: one in the last quarter of 2003 and another in the second quarter of 2011. The final model fits the observed data well (Figure S1). The longitudinal data analysis estimated an average cephalosporin consumption in the EU/EEA of 2.154 (SE 0.425) DDD per 1000 inhabitants per day in the first quarter of 1997, which did not change significantly over time: −0.003 (SE 0.006) DDD per 1000 inhabitants per day per quarter up to the last quarter of 2003; +0.006 (SE 0.013) DDD per 1000 inhabitants per day per quarter until the second quarter of 2011; and +0.009 (SE 0.015) DDD per 1000 inhabitants per day per quarter afterwards. Furthermore, the longitudinal data analysis showed significant seasonal variation with an amplitude of 0.616 (SE 0.09) DDD per 1000 inhabitants per day, which decreased significantly over time with 0.004 (SE 0.001) DDD per 1000 inhabitants per day per quarter (Figure 3). The seasonal variation in cephalosporin consumption is shown in Figures S2 and S3. Seasonal variations were observed in all countries.

Based on the fitted model, cephalosporin consumption in the community in 1997 was significantly higher than average in Belgium, Cyprus (total care data), Greece, Italy, Luxembourg and Portugal, and significantly lower than average in Denmark, Estonia, Germany, Iceland, Lithuania, the Netherlands, Slovenia, Sweden and the United Kingdom. The decrease in cephalosporin consumption between 1997 and the last quarter of 2003 was significantly larger than average in Belgium, Hungary and Spain, and smaller than average in Croatia and Slovakia. The increase in cephalosporin consumption between the first quarter of 2004 and the second quarter of 2011 was significantly larger than average in Germany, Lithuania and Slovakia. The increase in cephalosporin consumption between the third quarter of 2011 and 2017 was significantly larger than average in Lithuania and Slovakia. Seasonal variation was significantly larger than average in Belgium, Germany, Greece, Hungary, Italy, Luxembourg, Portugal and Slovakia, and significantly smaller than average in Denmark, Estonia, Finland, Iceland, Lithuania, the Netherlands, Slovenia, Sweden and the United Kingdom.

Between 2009 and 2017 cephalosporin consumption increased in 12 EU/EEA countries (Table S1). This increase was the highest in Bulgaria followed by Spain, Poland and Romania. Iceland, a country with low cephalosporin consumption in the community, has doubled its consumption since 2009, mainly due to an increase in the consumption of the first-generation cephalosporin cefalexin. As in 2009, the Netherlands and Denmark still had the lowest consumption of cephalosporins in the community in 2017. The Netherlands even showed a further decrease mainly due to the continuous decrease in the consumption of the second-generation cephalosporins (cefaclor and cefuroxime), but also the third-generation cephalosporin ceftibuten. Fourth-generation cephalosporins were not consumed in most northern EU/EEA countries, or Portugal, during the period 2009–2017.

### Compositional data analysis, 2009–2017

The proportional consumption of both second- and third-generation cephalosporins significantly increased over time relative to that of first- and fourth-generation cephalosporins. The proportional consumption of third-generation cephalosporins significantly increased relative to that of second-generation cephalosporins (Table 3).

**Table 3.** Change in the composition of the consumption of cephalosporins (ATC J01DB, J01DC, J01DD and J01DE) in the community, expressed in DDD (ATC/DDD index 2019) per 1000 inhabitants per day, 30 EU/EEA countries, as a function of time during 1997–2017

	J01DB	J01DC	J01DD	J01DE
J01DB		**−0.0702**	**−0.0995**	−0.0139
J01DC	**0.0702**		**−0.0293**	**0.0563**
J01DD	**0.0995**	**0.0293**		**0.0856**
J01DE	0.0139	**−0.0563**	**−0.0856**	

Values are estimated changes in the log ratio of the row versus column subgroup of antibiotics with increasing time. Bold type indicates a statistically significant effect; positive values represent an increase and negative values represent a decrease. J01DB, first-generation cephalosporins; J01DC, second-generation cephalosporins; J01DD, third-generation cephalosporins; J01DE, fourth-generation cephalosporins.

Trends of proportional consumption in individual countries are shown in Figure S4. When comparing the composition of the consumption of cephalosporins in 2017 with that in 2009, both increases and decreases were observed. For first-generation cephalosporins, changes were substantial, with increases >10% reported for Iceland (+58.37%), Sweden (+21.94%) and Ireland (+13.37%), and decreases >10% reported for Bulgaria (−33.72%), Latvia (−29.89%), Lithuania (−28.57%), Croatia (−13.64%), and Estonia (−12.33%). In addition, increases >5% were reported for three countries (Austria, the Netherlands and Slovenia) while decreases >5% were reported for five countries [Czechia (2015 data), Denmark, Norway, Portugal and Romania (total care data; coverage in 2009 limited to 30%–40%)]. The increase observed for the Netherlands was minimal in absolute value with consumption of first-generation cephalosporins consistently representing <0.01 DDD per 1000 inhabitants per day. Substantial decreases were observed due to the discontinuation of cefatrizine (J01DB07) in four countries and of cefradine (J01DB09) in nine countries. The changes in proportions of first-generation cephalosporins were matched by changes in proportions of either second-generation or third-generation cephalosporins. For second- and third-generation cephalosporins, decreases >5% were observed for 10 and 7 countries, respectively, while increases >5% were observed for 11 and 8 countries, respectively. The largest changes in the proportional consumption of second-generation cephalosporins were observed for Lithuania (+34.20%) and Latvia (+30.01%), and for Iceland (−59.39%). The largest changes in the proportional consumption of third-generation cephalosporins were observed for Romania (+22.11%; total care data; coverage in 2009 limited to 30%–40%) and Malta (+15.91%), and for Austria (−27.91%). Changes in the proportional consumption of fourth-generation cephalosporins were minimal and never exceeded 1%.

## Discussion

This study describes the consumption of cephalosporins in the community in the EU/EEA. The longitudinal data analysis showed no significant changes in the trend of cephalosporin consumption between 1997 and 2017. On the other hand, its seasonal variation decreased significantly over time. We also observed considerable variation in trends and composition over time at the country level. Overall, the significantly increasing consumption of second- and third-generation cephalosporins went hand-in-hand with a proportional decrease of first-generation cephalosporins in most countries. These trends had already been observed for the period 1997–2009.^4^

The proportional consumption of cephalosporins (a subgroup of other β-lactam antibacterials, ATC group J01D) out of all antibacterials for systemic use (J01) in 2017 ranged from 0.22% in Denmark to 23.96% in Greece.^5^ In 2017, consumption of cephalosporins (J01D) in European countries that are not part of the ESAC-Net but covered by the WHO Europe Antimicrobial Medicines Consumption (AMC) Network also showed considerable variation and ranged between 9.5% (Armenia) to 27.5% (Uzbekistan) of total, i.e. community and hospital sector combined, consumption of antibacterials for systemic use (J01).^9^

A remarkable gradient in cephalosporin consumption was observed in the EU/EEA, with a higher consumption and seasonal variation in southern and eastern countries than in northern countries. However, while the countries with the highest cephalosporin consumption in the community, i.e. Greece and Cyprus (total care data), decreased their consumption, it nearly doubled in Bulgaria and Romania (total care data; coverage in 2009 limited to 30%–40%). In Malta, after a considerable increase up to 2009, consumption of, mostly second-generation, cephalosporins has decreased since 2014. Furthermore, we observed a continuous decreasing trend of cephalosporin consumption in the community in several countries since 1997, in particular in Belgium, France and Italy. Denmark and the Netherlands remained the countries with the lowest cephalosporin consumption in the community.^4^^,^^5^

Since 1997, the consumption of first-generation cephalosporins in the community continued to decrease significantly, which could be explained by the decreased or discontinued consumption of cefadroxil, cefatrizine and cefradine, mainly in high cephalosporin consuming countries such as France, Greece, Luxembourg, and also in Portugal. The consumption of the older (first-generation) cephalosporins also continued to decrease in favour of the newer (second- and third-generation) cephalosporins in most countries. In 2017, second-generation cephalosporins were consumed twice as much compared with 1997, which was due to the four-fold increase of cefuroxime consumption. Observed increases in the proportional consumption of second- and third-generation cephalosporins are not only due to the decrease in the consumption of first-generation cephalosporins, but also due to the increase of the more extended-spectrum cephalosporins. Half of the EU/EEA countries showed an increased consumption of third-generation cephalosporins during 1997–2017. Fourth-generation cephalosporins were consumed in 16 countries.

In a recently published study based on the ESAC-Net data from the period 2001–2018, a decreasing trend was observed for both in the community and hospital consumption of third-generation cephalosporins in the EU/EEA. During the same period, third-generation cephalosporin resistance percentages in invasive *Escherichia coli* and *Klebsiella pneumoniae* isolates seemed to be stabilizing, thus suggesting an effect of recent public health efforts in promoting prudent antimicrobial use in the EU/EEA.^10^

The highest proportions of parenterally administered cephalosporins (mainly ceftriaxone) in the community were observed for France, Greece, Italy and Latvia. In Italy, consumption of parenteral ceftriaxone increased until 2013 and decreased steadily thereafter. Outpatient parenteral antimicrobial therapy (OPAT) is common in Italy.^11^

Although cephalosporins are also used for the treatment of urinary tract infections, and skin and soft tissue infections, the observed seasonal variation in cephalosporin consumption suggests that cephalosporins are mostly prescribed to treat seasonal respiratory infections, which are often viral in nature, and the prescriptions are therefore not following treatment guidelines.^12^^,^^13^ However, confirmation of this hypothesis would require information on the indication for cephalosporin prescriptions.

The substantial variations between countries and over time suggest that cephalosporins, in particular the more extended-spectrum cephalosporins such as third-generation cephalosporins, are to a large extent inappropriately used. In Bulgaria, a country with high consumption of third-generation cephalosporins in the community, the percentage of invasive isolates with acquired non-susceptibility to third-generation cephalosporins reached 41.0% in *E. coli* and 75.9% in *K. pneumoniae* in 2016.^10^ These resistance percentages are considerably higher than in countries such as Belgium, for which we observed a lower consumption of third-generation cephalosporins.^14^

With the exception of six cephalosporins that represented <0.05% of cephalosporin consumption in the EU/EEA, all second- and third-generation cephalosporins consumed in the EU/EEA are listed as Watch group antibiotics in the 2019 WHO Access, Watch or Reserve (AWaRe) classification list.^15^ The Watch group includes antibiotics that have higher resistance potential than Access group antibiotics and includes most of the highest priority agents among the Critically Important Antimicrobials for Human Medicine.^16^ In view of our observation that, in most EU/EEA countries, the absolute and proportional consumption of second- and third-generation cephalosporins significantly increased at the expense of the consumption of first-generation cephalosporins, all second- and third-generation cephalosporins should be prioritized as key targets of stewardship programmes and monitoring in these countries.

For a more-detailed discussion on the limitations of the collected data, we refer to the article on antibacterials for systemic use, included in this series.^6^ For a discussion on the limitations of the statistical approach used in this study and potential explanations for the common change-points detected through these analyses, we refer readers to the tutorial included in this series.^7^

In conclusion, the longitudinal and compositional trends of cephalosporin consumption in the community in EU/EEA countries during 1997–2017 continued to follow the same patterns as already reported for 1997–2009, namely a significant increase in consumption of the more extended-spectrum cephalosporins. Further research is needed to better understand the observed trends and seasonal variations. These results from ESAC-Net may be used as a reference to gauge future interventions to optimize cephalosporin consumption in the community in EU/EEA countries.

## Supplementary Material

dkab174_Supplementary_Data

## Supplementary data

Tables S1 and S2 and Figures S1 to S4 are available as Supplementary data at *JAC* Online.
