# Supplementary material for: Consumption of cephalosporins in the community, European Union/European Economic Area, 1997–2017
Source: J Antimicrob Chemother. 2021 Aug 1;76(Suppl 2):ii22–9. doi: 10.1093/jac/dkab174 (PMC8314097; doi:10.1093/jac/dkab174)
Supplement: dkab174_Supplementary_Data [file dkab174_supplementary_data.docx]

**Supplementary data**

**Table S1. Consumption of cephalosporins (ATC J01DB, J01DC, J01DD and J01DE) in the community, expressed in DDD (ATC/DDD index 2019) per 1000 inhabitants per day, 30 EU/EEA countries, 1997-2017.**

| **Country** | **1997** | **1998** | **1999** | **2000** | **2001** | **2002** | **2003** | **2004** | **2005** | **2006** | **2007** | **2008** | **2009** | **2010** | **2011** | **2012** | **2013** | **2014** | **2015** | **2016** | **2017** |
| --- | --- | --- | --- | --- | --- | --- | --- | --- | --- | --- | --- | --- | --- | --- | --- | --- | --- | --- | --- | --- | --- |
| **Austria** | **-** | **1.58** | **1.73** | **1.49** | **1.39** | **1.39** | **1.63** | **1.56** | **1.67** | **1.57** | **1.70** | **1.70** | **1.80** | **1.70** | **1.66** | **1.58** | **1.95** | **1.48** | **1.44** | **1.41** | **1.51** |
| DB | **-** | 0.32 | 0.31 | 0.29 | 0.29 | 0.29 | 0.31 | 0.29 | 0.29 | 0.29 | 0.29 | 0.30 | 0.31 | 0.30 | 0.31 | 0.28 | 0.34 | 0.31 | 0.31 | 0.31 | 0.34 |
| DC | **-** | 0.53 | 0.64 | 0.50 | 0.39 | 0.35 | 0.46 | 0.50 | 0.61 | 0.57 | 0.61 | 0.64 | 0.71 | 0.70 | 0.72 | 0.72 | 0.99 | 0.88 | 0.90 | 0.88 | 0.94 |
| DD | **-** | 0.73 | 0.79 | 0.70 | 0.71 | 0.75 | 0.86 | 0.77 | 0.77 | 0.71 | 0.79 | 0.76 | 0.79 | 0.70 | 0.64 | 0.58 | 0.63 | 0.30 | 0.23 | 0.21 | 0.24 |
| DE | **-** | **-** | **-** | **-** | <0.01 | **-** | **-** | **-** | **-** | **-** | **-** | **-** | **-** | **-** | <0.01 | <0.01 | <0.01 | <0.01 | **-** | <0.01 | <0.01 |
| **Belgium** | **3.97** | **4.41** | **4.20** | **3.88** | **3.18** | **3.38** | **3.27** | **3.14** | **3.05** | **2.58** | **2.36** | **2.02** | **1.82** | **1.59** | **1.52** | **1.53** | **1.53** | **1.42** | **1.44** | **1.32** | **1.17** |
| DB | 1.00 | 0.89 | 0.67 | 0.53 | 0.40 | 0.33 | 0.27 | 0.23 | 0.18 | 0.16 | 0.15 | 0.16 | 0.15 | 0.13 | 0.12 | 0.12 | 0.11 | 0.10 | 0.10 | 0.08 | 0.05 |
| DC | 2.97 | 3.52 | 3.52 | 3.35 | 2.78 | 3.05 | 2.99 | 2.91 | 2.86 | 2.41 | 2.21 | 1.87 | 1.67 | 1.47 | 1.40 | 1.41 | 1.42 | 1.32 | 1.34 | 1.24 | 1.12 |
| DD | <0.01 | <0.01 | <0.01 | <0.01 | <0.01 | <0.01 | <0.01 | <0.01 | <0.01 | <0.01 | <0.01 | <0.01 | <0.01 | <0.01 | <0.01 | <0.01 | <0.01 | <0.01 | <0.01 | <0.01 | <0.01 |
| DE | **-** | **-** | **-** | **-** | **-** | **-** | <0.01 | **-** | **-** | **-** | **-** | **-** | <0.01 | **-** | **-** | **-** | **-** | **-** | **-** | **-** | **-** |
| **Bulgaria** | **-** | **-** | ***1.38*** | ***2.52*** | ***2.50*** | ***1.43*** | ***1.38*** | ***1.68*** | ***2.07*** | **1.70** | **1.87** | **2.08** | **2.30** | **2.37** | **2.59** | **2.44** | **2.80** | **3.38** | **3.89** | **3.84** | **4.11** |
| DB | **-** | **-** | *1.21* | *1.58* | *2.08* | *0.88* | *1.01* | *1.16* | *1.45* | 1.17 | 1.12 | 1.04 | 0.83 | 0.72 | 0.69 | 0.50 | 0.50 | 0.38 | 0.14 | 0.11 | 0.09 |
| DC | **-** | **-** | *0.11* | *0.59* | *0.34* | *0.34* | *0.23* | *0.36* | *0.47* | 0.50 | 0.70 | 0.93 | 1.31 | 1.40 | 1.53 | 1.51 | 1.71 | 2.26 | 3.01 | 2.94 | 3.13 |
| DD | **-** | **-** | *0.06* | *0.35* | *0.07* | *0.19* | *0.14* | *0.12* | *0.14* | 0.03 | 0.05 | 0.12 | 0.17 | 0.25 | 0.36 | 0.43 | 0.58 | 0.74 | 0.74 | 0.80 | 0.89 |
| DE | **-** | **-** | *<0.01* | *0.01* | *0.01* | *0.01* | *0.01* | *0.03* | *0.01* | <0.01 | <0.01 | <0.01 | <0.01 | <0.01 | <0.01 | <0.01 |  | <0.01 | <0.01 |  |  |
| **Croatia** | **-** | **-** | **-** | **-** | **3.25** | **3.77** | **4.00** | **3.53** | **3.74** | **3.43** | **3.71** | **4.11** | **3.91** | **3.45** | **2.87** | **3.53** | **2.99** | **2.81** | **2.74** | **2.49** | **2.47** |
| DB | **-** | **-** | **-** | **-** | 1.71 | 2.09 | 2.14 | 1.91 | 1.89 | 1.71 | 1.91 | 1.65 | 1.28 | 1.05 | 0.84 | 0.79 | 0.77 | 0.72 | 0.66 | 0.60 | 0.47 |
| DC | **-** | **-** | **-** | **-** | 1.15 | 1.32 | 1.40 | 1.22 | 1.42 | 1.28 | 1.11 | 1.72 | 1.77 | 1.59 | 1.26 | 1.95 | 1.77 | 1.85 | 1.85 | 1.69 | 1.67 |
| DD | **-** | **-** | **-** | **-** | 0.39 | 0.36 | 0.45 | 0.40 | 0.43 | 0.44 | 0.69 | 0.74 | 0.86 | 0.82 | 0.77 | 0.79 | 0.45 | 0.24 | 0.23 | 0.20 | 0.33 |
| DE | **-** | **-** | **-** | **-** | **-** | **-** | **-** | <0.01 | <0.01 | **-** | **-** | **-** | **-** | **-** | **-** | **-** | **-** | **-** | **-** | **-** | **-** |
| **Cyprus** | **-** | **-** | **-** | **-** | **-** | **-** | **-** | **-** | **-** | ***6.20*** | ***6.95*** | ***6.68*** | ***6.38*** | ***5.33*** | ***5.99*** | ***5.27*** | ***4.75*** | ***4.37*** | ***5.14*** | ***5.05*** | ***5.69*** |
| DB | **-** | **-** | **-** | **-** | **-** | **-** | **-** | **-** | **-** | *0.67* | *0.66* | *0.59* | *0.47* | *0.43* | *0.39* | *0.37* | *0.28* | *0.23* | *0.28* | *0.23* | *0.25* |
| DC | **-** | **-** | **-** | **-** | **-** | **-** | **-** | **-** | **-** | *5.09* | *5.69* | *5.50* | *5.32* | *4.39* | *4.85* | *4.19* | *3.72* | *3.36* | *4.03* | *3.86* | *4.41* |
| DD | **-** | **-** | **-** | **-** | **-** | **-** | **-** | **-** | **-** | *0.45* | *0.59* | *0.58* | *0.58* | *0.50* | *0.75* | *0.71* | *0.75* | *0.78* | *0.83* | *0.96* | *1.03* |
| DE | **-** | **-** | **-** | **-** | **-** | **-** | **-** | **-** | **-** | ***-*** | ***-*** | ***-*** | ***-*** | ***-*** | ***-*** | ***-*** | *<0.01* | *<0.01* | *<0.01* | *<0.01* | *<0.01* |
| **Czech Republic** | **-** | **1.35** | **1.26** | **-** | **-** | **-** | **1.00** | **0.96** | **1.19** | **0.91** | **1.05** | **1.36** | **1.52** | **1.59** | **1.47** | **1.41** | **1.72** | **1.99** | **2.17** | **-** | **-** |
| DB | **-** | 0.51 | 0.43 | **-** | **-** | **-** | 0.24 | 0.18 | 0.17 | 0.11 | 0.10 | 0.13 | 0.14 | 0.13 | 0.12 | 0.10 | 0.09 | 0.09 | 0.09 | **-** | **-** |
| DC | **-** | 0.83 | 0.83 | **-** | **-** | **-** | 0.75 | 0.77 | 1.01 | 0.80 | 0.95 | 1.15 | 1.30 | 1.38 | 1.27 | 1.24 | 1.56 | 1.82 | 1.99 | **-** | **-** |
| DD | **-** | 0.01 | <0.01 | **-** | **-** | **-** | <0.01 | <0.01 | <0.01 | <0.01 | <0.01 | 0.09 | 0.07 | 0.08 | 0.08 | 0.06 | 0.06 | 0.08 | 0.08 | **-** | **-** |
| DE | **-** | **-** | **-** | **-** | **-** | **-** | **-** | **-** | **-** | **-** | **-** | <0.01 | <0.01 | <0.01 | <0.01 | <0.01 | <0.01 | <0.01 | <0.01 | **-** | **-** |
| Country, community consumption of cephalosporins (J01DB, J01DC, J01DD and J01DE combined); DB, first-generation cephalosporins (J01DB); DC, second-generation cephalosporins (J01DC); DD, third-generation cephalosporins (J01DD); DE, fourth-generation cephalosporins (J01DE); **-**, no consumption reported; Numbers reported in *italic* are total care data, i.e. community and hospital sector combined; ^a^Data for Romania have a coverage in 2009 limited to 30-40%; ^b^Data for Spain include private prescriptions as of 2016. | | | | | | | | | | | | | | | | | | | | | |
| **Denmark** | **0.02** | **0.03** | **0.02** | **0.02** | **0.03** | **0.03** | **0.02** | **0.02** | **0.03** | **0.03** | **0.03** | **0.03** | **0.03** | **0.03** | **0.05** | **0.03** | **0.03** | **0.03** | **0.03** | **0.03** | **0.03** |
| DB | <0.01 | <0.01 | <0.01 | <0.01 | 0.01 | 0.01 | 0.01 | 0.01 | 0.01 | 0.01 | 0.01 | 0.01 | 0.01 | 0.01 | 0.01 | 0.01 | 0.01 | <0.01 | <0.01 | <0.01 | <0.01 |
| DC | 0.02 | 0.02 | 0.02 | 0.02 | 0.02 | 0.02 | 0.02 | 0.02 | 0.02 | 0.02 | 0.02 | 0.02 | 0.02 | 0.02 | 0.04 | 0.02 | 0.02 | 0.02 | 0.02 | 0.02 | 0.02 |
| DD | <0.01 | <0.01 | <0.01 | <0.01 | <0.01 | <0.01 | <0.01 | <0.01 | <0.01 | <0.01 | <0.01 | <0.01 | 0.01 | <0.01 | <0.01 | 0.01 | <0.01 | <0.01 | <0.01 | 0.01 | 0.01 |
| DE | **-** | **-** | **-** | **-** | **-** | **-** | **-** | **-** | **-** | **-** | **-** | **-** | **-** | **-** | **-** | **-** | **-** | **-** | **-** | **-** | **-** |
| **Estonia** | **-** | **-** | **-** | **-** | ***0.68*** | **0.58** | **0.60** | **0.66** | **0.71** | **0.78** | **0.81** | **0.85** | **0.83** | **0.89** | **0.98** | **0.99** | **1.10** | **1.14** | **1.22** | **1.16** | **1.18** |
| DB | **-** | **-** | **-** | **-** | *0.36* | 0.31 | 0.33 | 0.33 | 0.33 | 0.32 | 0.25 | 0.26 | 0.22 | 0.22 | 0.24 | 0.23 | 0.23 | 0.24 | 0.21 | 0.15 | 0.17 |
| DC | **-** | **-** | **-** | **-** | *0.30* | 0.27 | 0.27 | 0.33 | 0.39 | 0.46 | 0.56 | 0.59 | 0.60 | 0.67 | 0.75 | 0.76 | 0.86 | 0.89 | 0.99 | 1.00 | 1.01 |
| DD | **-** | **-** | **-** | **-** | *0.02* | <0.01 | <0.01 | <0.01 | <0.01 | <0.01 | <0.01 | <0.01 | <0.01 | <0.01 | <0.01 | <0.01 | <0.01 | 0.01 | 0.01 | <0.01 | <0.01 |
| DE | **-** | **-** | **-** | **-** | *<0.01* | **-** | **-** | **-** | **-** | **-** | **-** | **-** | **-** | **-** | **-** | **-** | **-** | **-** | **-** | **-** | **-** |
| **Finland** | **2.26** | **2.13** | **2.24** | **2.24** | **2.32** | **2.27** | **2.29** | **2.13** | **2.21** | **2.21** | **2.38** | **2.32** | **2.32** | **2.33** | **2.36** | **2.30** | **2.28** | **2.25** | **2.10** | **2.05** | **1.94** |
| DB | 1.80 | 1.80 | 1.95 | 1.99 | 2.09 | 2.08 | 2.12 | 2.00 | 2.08 | 2.11 | 2.28 | 2.24 | 2.24 | 2.24 | 2.27 | 2.23 | 2.22 | 2.19 | 2.05 | 2.00 | 1.90 |
| DC | 0.44 | 0.32 | 0.29 | 0.24 | 0.23 | 0.19 | 0.17 | 0.13 | 0.13 | 0.10 | 0.09 | 0.09 | 0.09 | 0.09 | 0.09 | 0.07 | 0.06 | 0.06 | 0.05 | 0.04 | 0.03 |
| DD | 0.02 | 0.01 | 0.01 | <0.01 | <0.01 | <0.01 | <0.01 | <0.01 | <0.01 | <0.01 | <0.01 | <0.01 | <0.01 | <0.01 | <0.01 | <0.01 | <0.01 | <0.01 | <0.01 | <0.01 | <0.01 |
| DE | **-** | **-** | **-** | **-** | **-** | **-** | **-** | **-** | **-** | **-** | **-** | **-** | **-** | **-** | **-** | **-** | **-** | **-** | **-** | **-** | **-** |
| **France** | **4.66** | **4.55** | **4.82** | **4.56** | **4.24** | **3.69** | **3.33** | **3.06** | **3.21** | **2.76** | **2.96** | **2.54** | **2.91** | **2.67** | **2.55** | **2.39** | **2.24** | **2.05** | **2.12** | **1.94** | **1.60** |
| DB | 1.90 | 1.70 | 1.75 | 1.23 | 0.85 | 0.58 | 0.38 | 0.26 | 0.22 | 0.17 | 0.12 | 0.09 | 0.09 | 0.07 | 0.06 | 0.05 | 0.05 | 0.04 | 0.03 | 0.03 | 0.02 |
| DC | 1.66 | 1.63 | 1.75 | 1.75 | 1.69 | 1.42 | 1.31 | 1.26 | 1.27 | 0.98 | 0.96 | 0.72 | 0.93 | 0.79 | 0.59 | 0.66 | 0.62 | 0.51 | 0.48 | 0.43 | 0.34 |
| DD | 1.10 | 1.22 | 1.32 | 1.59 | 1.70 | 1.69 | 1.64 | 1.55 | 1.71 | 1.62 | 1.87 | 1.72 | 1.90 | 1.81 | 1.89 | 1.67 | 1.58 | 1.50 | 1.60 | 1.48 | 1.23 |
| DE | **-** | **-** | **-** | **-** | **-** | **-** | **-** | **-** | **-** | **-** | <0.01 | <0.01 | <0.01 | <0.01 | <0.01 | <0.01 | <0.01 | <0.01 | <0.01 | <0.01 | <0.01 |
| **Germany** | **0.94** | **1.03** | **0.95** | **0.93** | **0.86** | **0.89** | **1.21** | **1.26** | **1.46** | **1.36** | **1.59** | **1.93** | **2.39** | **2.61** | **2.72** | **2.81** | **3.22** | **3.01** | **3.11** | **3.03** | **2.81** |
| DB | 0.17 | 0.18 | 0.17 | 0.17 | 0.15 | 0.14 | 0.13 | 0.12 | 0.12 | 0.12 | 0.11 | 0.11 | 0.09 | 0.09 | 0.08 | 0.07 | 0.07 | 0.07 | 0.05 | 0.05 | 0.05 |
| DC | 0.38 | 0.39 | 0.36 | 0.34 | 0.33 | 0.37 | 0.70 | 0.76 | 0.93 | 0.89 | 1.09 | 1.39 | 1.79 | 2.02 | 2.16 | 2.30 | 2.72 | 2.63 | 2.72 | 2.67 | 2.52 |
| DD | 0.39 | 0.45 | 0.42 | 0.42 | 0.39 | 0.39 | 0.37 | 0.37 | 0.41 | 0.34 | 0.39 | 0.42 | 0.51 | 0.50 | 0.48 | 0.44 | 0.43 | 0.32 | 0.33 | 0.31 | 0.25 |
| DE | **-** | **-** | **-** | **-** | **-** | **-** | **-** | **-** | **-** | **-** | **-** | **-** | **-** | **-** | **-** | **-** | **-** | **-** | **-** | **-** | **-** |
| **Greece** | **6.26** | **6.06** | **6.16** | **6.68** | **6.64** | **6.73** | **6.21** | ***7.08*** | ***7.70*** | ***7.67*** | ***8.83*** | ***9.40*** | **8.65** | ***8.88*** | **7.70** | **6.54** | **7.37** | **7.26** | **7.47** | **7.38** | **7.65** |
| DB | 2.33 | 1.45 | 1.40 | 0.94 | 0.65 | 0.50 | 0.37 | *0.30* | *0.23* | *0.19* | *0.19* | *0.09* | 0.06 | *<0.01* | 0.01 | <0.01 | <0.01 | 0.01 | <0.01 | 0.01 | <0.01 |
| DC | 3.86 | 4.52 | 4.71 | 5.70 | 5.93 | 6.16 | 5.77 | *6.55* | *7.27* | *7.18* | *8.10* | *8.84* | 8.29 | *8.63* | 7.59 | 6.45 | 7.27 | 7.12 | 7.35 | 7.12 | 7.46 |
| DD | 0.07 | 0.09 | 0.05 | 0.04 | 0.05 | 0.07 | 0.07 | *0.22* | *0.20* | *0.29* | *0.53* | *0.45* | 0.29 | *0.23* | 0.10 | 0.08 | 0.10 | 0.13 | 0.11 | 0.24 | 0.18 |
| DE | <0.01 | <0.01 | <0.01 | <0.01 | <0.01 | <0.01 | <0.01 | *0.01* | *0.01* | *0.01* | *0.01* | *0.01* | <0.01 | *0.01* | <0.01 | <0.01 | <0.01 | <0.01 | <0.01 | 0.01 | 0.01 |
| **Hungary** | **-** | **2.52** | **3.35** | **2.53** | **2.43** | **2.09** | **2.26** | **2.20** | **2.32** | **2.09** | **1.64** | **1.86** | **1.98** | **1.92** | **1.91** | **1.77** | **1.81** | **1.87** | **1.98** | **2.06** | **2.10** |
| DB | **-** | 0.38 | 0.40 | 0.24 | 0.20 | 0.16 | 0.13 | 0.11 | 0.09 | 0.07 | 0.05 | 0.04 | 0.04 | 0.03 | 0.02 | 0.01 | 0.01 | 0.01 | 0.01 | 0.01 | 0.01 |
| DC | **-** | 1.91 | 2.51 | 1.89 | 1.77 | 1.51 | 1.69 | 1.66 | 1.75 | 1.55 | 1.23 | 1.43 | 1.56 | 1.51 | 1.52 | 1.42 | 1.45 | 1.61 | 1.76 | 1.75 | 1.79 |
| DD | **-** | 0.23 | 0.44 | 0.40 | 0.46 | 0.42 | 0.44 | 0.44 | 0.49 | 0.47 | 0.36 | 0.38 | 0.39 | 0.39 | 0.37 | 0.34 | 0.35 | 0.26 | 0.21 | 0.30 | 0.31 |
| DE | **-** | **-** | **-** | **-** | **-** | **-** | **-** | **-** | **-** | **-** | **-** | **-** | **-** | **-** | **-** | **-** | **-** | **-** | **-** | **-** | **-** |
| Country, community consumption of cephalosporins (J01DB, J01DC, J01DD and J01DE combined); DB, first-generation cephalosporins (J01DB); DC, second-generation cephalosporins (J01DC); DD, third-generation cephalosporins (J01DD); DE, fourth-generation cephalosporins (J01DE); **-**, no consumption reported; Numbers reported in *italic* are total care data, i.e. community and hospital sector combined;  ^a^Data for Romania have a coverage in 2009 limited to 30-40%; ^b^Data for Spain include private prescriptions as of 2016. | | | | | | | | | | | | | | | | | | | | | |
| **Iceland** | ***0.55*** | ***0.55*** | ***0.57*** | ***0.56*** | ***0.51*** | ***0.53*** | ***0.48*** | ***0.42*** | ***0.50*** | **0.19** | **0.10** | **0.26** | **0.30** | ***0.59*** | ***0.60*** | ***0.66*** | ***0.74*** | **0.46** | **0.46** | **0.51** | **0.61** |
| DB | *0.11* | *0.09* | *0.10* | *0.12* | *0.11* | *0.14* | *0.14* | *0.17* | *0.18* | 0.08 | 0.10 | 0.17 | 0.12 | *0.36* | *0.35* | *0.44* | *0.51* | 0.42 | 0.45 | 0.50 | 0.60 |
| DC | *0.40* | *0.41* | *0.42* | *0.39* | *0.35* | *0.34* | *0.28* | *0.20* | *0.25* | 0.11 | **-** | 0.09 | 0.18 | *0.18* | *0.18* | *0.16* | *0.14* | 0.04 | 0.01 | <0.01 | <0.01 |
| DD | *0.05* | *0.04* | *0.04* | *0.05* | *0.06* | *0.05* | *0.05* | *0.06* | *0.07* | <0.01 | **-** | **-** | **-** | *0.06* | *0.06* | *0.06* | *0.08* | **-** | <0.01 | 0.01 | 0.01 |
| DE | ***-*** | ***-*** | ***-*** | ***-*** | ***-*** | ***-*** | ***-*** | ***-*** | ***-*** | **-** | **-** | **-** | **-** | ***-*** | ***-*** | ***-*** | ***-*** | **-** | **-** | **-** | **-** |
| **Ireland** | **-** | **1.72** | **1.97** | **1.90** | **2.01** | **1.89** | **2.00** | **1.91** | **1.82** | **1.87** | **1.96** | **1.56** | **1.32** | **1.21** | **1.21** | **1.24** | **1.36** | **1.10** | **1.16** | **1.17** | **1.08** |
| DB | **-** | 0.41 | 0.41 | 0.37 | 0.37 | 0.34 | 0.35 | 0.33 | 0.30 | 0.30 | 0.30 | 0.30 | 0.28 | 0.19 | 0.17 | 0.18 | 0.20 | 0.25 | 0.28 | 0.33 | 0.37 |
| DC | **-** | 1.13 | 1.36 | 1.35 | 1.48 | 1.40 | 1.49 | 1.43 | 1.42 | 1.47 | 1.54 | 1.14 | 0.95 | 0.92 | 0.94 | 0.96 | 1.07 | 0.79 | 0.84 | 0.80 | 0.68 |
| DD | **-** | 0.18 | 0.20 | 0.18 | 0.17 | 0.16 | 0.16 | 0.15 | 0.09 | 0.10 | 0.12 | 0.12 | 0.10 | 0.10 | 0.10 | 0.10 | 0.09 | 0.07 | 0.04 | 0.04 | 0.03 |
| DE | **-** | **-** | **-** | **-** | **-** | **-** | **-** | **-** | **-** | **-** | **-** | **-** | **-** | **-** | **-** | **-** | **-** | **-** | **-** | **-** | **-** |
| **Italy** | **-** | **-** | **3.87** | **3.65** | **3.67** | **3.42** | **3.35** | **3.08** | **3.10** | **2.88** | **2.72** | **2.77** | **2.77** | **2.60** | **2.53** | **2.36** | **2.48** | **2.34** | **2.33** | **2.26** | **1.94** |
| DB | **-** | **-** | 0.26 | 0.22 | 0.21 | 0.18 | 0.17 | 0.16 | 0.14 | 0.13 | 0.13 | 0.13 | 0.12 | 0.11 | 0.09 | 0.08 | 0.08 | 0.08 | 0.07 | 0.07 | 0.06 |
| DC | **-** | **-** | 1.89 | 1.75 | 1.55 | 1.36 | 1.23 | 1.10 | 0.99 | 0.90 | 0.76 | 0.68 | 0.60 | 0.50 | 0.46 | 0.39 | 0.36 | 0.31 | 0.28 | 0.26 | 0.20 |
| DD | **-** | **-** | 1.71 | 1.67 | 1.88 | 1.87 | 1.94 | 1.82 | 1.96 | 1.84 | 1.83 | 1.95 | 2.05 | 1.98 | 1.97 | 1.88 | 2.03 | 1.95 | 1.97 | 1.93 | 1.68 |
| DE | **-** | **-** | 0.02 | 0.01 | 0.02 | 0.01 | 0.01 | 0.01 | 0.01 | 0.01 | <0.01 | <0.01 | <0.01 | <0.01 | <0.01 | <0.01 | <0.01 | <0.01 | <0.01 | <0.01 | <0.01 |
| **Latvia** | **-** | **-** | **-** | **-** | **-** | **0.41** | **-** | **0.33** | **0.43** | **0.54** | **0.59** | **0.51** | **0.45** | **0.57** | **0.50** | **0.47** | **0.52** | **0.48** | **0.52** | **0.57** | **0.66** |
| DB | **-** | **-** | **-** | **-** | **-** | 0.28 | **-** | 0.22 | 0.28 | 0.33 | 0.35 | 0.27 | 0.24 | 0.33 | 0.27 | 0.19 | 0.20 | 0.17 | 0.18 | 0.16 | 0.15 |
| DC | **-** | **-** | **-** | **-** | **-** | 0.11 | **-** | 0.10 | 0.12 | 0.14 | 0.19 | 0.20 | 0.16 | 0.18 | 0.19 | 0.23 | 0.27 | 0.28 | 0.30 | 0.34 | 0.44 |
| DD | **-** | **-** | **-** | **-** | **-** | 0.02 | **-** | 0.01 | 0.03 | 0.07 | 0.05 | 0.04 | 0.05 | 0.06 | 0.03 | 0.04 | 0.04 | 0.03 | 0.04 | 0.07 | 0.07 |
| DE | **-** | **-** | **-** | **-** | **-** | <0.01 | **-** | <0.01 | **-** | **-** | **-** | **-** | **-** | **-** | **-** | **-** | **-** | **-** | **-** | **-** | **-** |
| **Lithuania** | **-** | **-** | **-** | **-** | **-** | **-** | **-** | **-** | **-** | ***0.87*** | ***1.80*** | ***3.19*** | ***1.26*** | ***1.08*** | ***1.31*** | **0.92** | **1.21** | **1.07** | **1.16** | **1.23** | **1.29** |
| DB | **-** | **-** | **-** | **-** | **-** | **-** | **-** | **-** | **-** | *0.77* | *1.32* | *2.24* | *0.62* | *0.39* | *0.44* | 0.21 | 0.27 | 0.25 | 0.26 | 0.24 | 0.27 |
| DC | **-** | **-** | **-** | **-** | **-** | **-** | **-** | **-** | **-** | *0.06* | *0.36* | *0.71* | *0.55* | *0.60* | *0.76* | 0.70 | 0.93 | 0.80 | 0.89 | 0.98 | 1.01 |
| DD | **-** | **-** | **-** | **-** | **-** | **-** | **-** | **-** | **-** | *0.04* | *0.12* | *0.24* | *0.09* | *0.08* | *0.11* | 0.01 | 0.01 | 0.01 | 0.01 | 0.01 | 0.02 |
| DE | **-** | **-** | **-** | **-** | **-** | **-** | **-** | **-** | **-** | *<0.01* | *<0.01* | *<0.01* | *<0.01* | *<0.01* | *<0.01* | **-** | **-** | **-** | **-** | <0.01 | **-** |
| **Luxembourg** | **5.26** | **5.39** | **5.78** | **5.38** | **5.15** | **5.11** | **5.43** | **4.74** | **5.03** | **4.25** | **4.47** | **4.24** | **4.33** | **4.04** | **3.80** | **3.62** | **3.80** | **3.39** | **3.46** | **3.38** | **2.36** |
| DB | 1.74 | 1.66 | 1.39 | 1.14 | 0.87 | 0.67 | 0.61 | 0.45 | 0.36 | 0.18 | 0.16 | 0.14 | 0.13 | 0.13 | 0.12 | 0.12 | 0.11 | 0.11 | 0.12 | 0.07 | 0.02 |
| DC | 3.51 | 3.72 | 4.39 | 4.23 | 4.27 | 4.42 | 4.80 | 4.28 | 4.66 | 4.06 | 4.30 | 4.08 | 4.18 | 3.89 | 3.67 | 3.49 | 3.67 | 3.27 | 3.33 | 3.30 | 1.99 |
| DD | 0.01 | 0.01 | 0.01 | 0.01 | 0.01 | 0.01 | 0.01 | 0.01 | 0.01 | 0.01 | 0.01 | 0.01 | 0.01 | 0.01 | 0.01 | 0.01 | 0.01 | 0.02 | 0.02 | 0.02 | 0.35 |
| DE | **-** | **-** | **-** | **-** | <0.01 | <0.01 | <0.01 | **-** | **-** | **-** | **-** | **-** | **-** | **-** | **-** | **-** | **-** | **-** | **-** | **-** | **-** |
| **Malta** | **-** | **-** | **-** | **-** | **-** | **-** | **-** | **-** | **-** | **-** | **2.97** | **4.90** | **5.50** | **5.04** | **5.68** | **5.27** | **5.52** | **4.57** | **4.11** | **3.57** | **3.02** |
| DB | **-** | **-** | **-** | **-** | **-** | **-** | **-** | **-** | **-** | **-** | 0.08 | 0.04 | 0.05 | 0.03 | 0.04 | 0.05 | 0.04 | 0.04 | 0.04 | 0.03 | 0.05 |
| DC | **-** | **-** | **-** | **-** | **-** | **-** | **-** | **-** | **-** | **-** | 2.80 | 4.71 | 5.27 | 4.79 | 5.36 | 4.92 | 5.07 | 3.58 | 3.33 | 2.93 | 2.40 |
| DD | **-** | **-** | **-** | **-** | **-** | **-** | **-** | **-** | **-** | **-** | 0.08 | 0.16 | 0.18 | 0.22 | 0.28 | 0.30 | 0.41 | 0.95 | 0.74 | 0.62 | 0.58 |
| DE | **-** | **-** | **-** | **-** | **-** | **-** | **-** | **-** | **-** | **-** | **-** | **-** | **-** | **-** | **-** | **-** | **-** | **-** | **-** | **-** | **-** |
| Country, community consumption of cephalosporins (J01DB, J01DC, J01DD and J01DE combined); DB, first-generation cephalosporins (J01DB); DC, second-generation cephalosporins (J01DC); DD, third-generation cephalosporins (J01DD); DE, fourth-generation cephalosporins (J01DE); **-**, no consumption reported; Numbers reported in *italic* are total care data, i.e. community and hospital sector combined; ^a^Data for Romania have a coverage in 2009 limited to 30-40%; ^b^Data for Spain include private prescriptions as of 2016. | | | | | | | | | | | | | | | | | | | | | |
| **Netherlands** | **0.13** | **0.11** | **0.10** | **0.08** | **0.07** | **0.07** | **0.06** | **0.05** | **0.05** | **0.05** | **0.05** | **0.04** | **0.04** | **0.04** | **0.04** | **0.04** | **0.04** | **0.03** | **0.03** | **0.03** | **0.03** |
| DB | 0.02 | 0.02 | 0.02 | 0.02 | 0.02 | 0.02 | 0.01 | 0.01 | 0.01 | 0.01 | 0.01 | <0.01 | <0.01 | <0.01 | <0.01 | <0.01 | <0.01 | <0.01 | <0.01 | <0.01 | <0.01 |
| DC | 0.09 | 0.07 | 0.05 | 0.05 | 0.04 | 0.04 | 0.03 | 0.03 | 0.03 | 0.03 | 0.03 | 0.03 | 0.03 | 0.03 | 0.03 | 0.03 | 0.03 | 0.02 | 0.02 | 0.02 | 0.02 |
| DD | 0.02 | 0.02 | 0.02 | 0.02 | 0.01 | 0.01 | 0.01 | 0.01 | 0.01 | 0.01 | 0.01 | 0.01 | 0.01 | 0.01 | 0.01 | 0.01 | 0.01 | 0.01 | 0.01 | 0.01 | <0.01 |
| DE | **-** | **-** | **-** | **-** | **-** | **-** | **-** | **-** | **-** | **-** | **-** | **-** | **-** | **-** | **-** | **-** | **-** | **-** | **-** | **-** | **-** |
| **Norway** | **-** | **0.23** | **-** | **-** | **0.25** | **0.27** | **0.29** | **0.28** | **0.23** | **0.16** | **0.16** | **0.14** | **0.13** | **0.12** | **0.11** | **0.11** | **0.11** | **0.09** | **0.08** | **0.07** | **0.06** |
| DB | **-** | 0.23 | **-** | **-** | 0.25 | 0.27 | 0.29 | 0.28 | 0.23 | 0.15 | 0.15 | 0.14 | 0.12 | 0.11 | 0.11 | 0.10 | 0.10 | 0.08 | 0.07 | 0.06 | 0.06 |
| DC | **-** | **-** | **-** | **-** | **-** | **-** | **-** | **-** | **-** | <0.01 | <0.01 | <0.01 | <0.01 | <0.01 | <0.01 | <0.01 | <0.01 | <0.01 | <0.01 | <0.01 | <0.01 |
| DD | **-** | **-** | **-** | **-** | **-** | **-** | **-** | **-** | **-** | <0.01 | <0.01 | <0.01 | <0.01 | <0.01 | <0.01 | 0.01 | 0.01 | 0.01 | 0.01 | 0.01 | 0.01 |
| DE | **-** | **-** | **-** | **-** | **-** | **-** | **-** | **-** | **-** | **-** | **-** | **-** | **-** | **-** | **-** | **-** | **-** | **-** | **-** | **-** | **-** |
| **Poland** | **-** | **2.11** | **2.10** | **2.05** | **2.29** | **2.06** | **-** | **2.53** | **1.72** | **-** | **2.04** | **2.21** | **2.89** | **2.45** | **2.59** | **2.20** | **2.50** | **2.37** | **2.87** | **2.75** | **3.99** |
| DB | **-** | 0.71 | 0.66 | 0.62 | 0.70 | 0.47 | **-** | 0.39 | 0.26 | **-** | 0.17 | 0.14 | 0.15 | 0.13 | 0.11 | 0.10 | 0.12 | 0.12 | 0.13 | 0.12 | 0.24 |
| DC | **-** | 1.36 | 1.38 | 1.38 | 1.56 | 1.56 | **-** | 2.14 | 1.46 | **-** | 1.88 | 2.07 | 2.74 | 2.32 | 2.48 | 2.09 | 2.37 | 2.24 | 2.72 | 2.62 | 3.74 |
| DD | **-** | 0.05 | 0.05 | 0.05 | 0.04 | 0.03 | **-** | <0.01 | <0.01 | **-** | <0.01 | <0.01 | <0.01 | <0.01 | <0.01 | 0.01 | 0.01 | 0.01 | 0.02 | 0.02 | 0.02 |
| DE | **-** |  | <0.01 | <0.01 | <0.01 | <0.01 | **-** | **-** | **-** | **-** | **-** | **-** | **-** | **-** | **-** | **-** | **-** | **-** | **-** | **-** | **-** |
| **Portugal** | **3.30** | **3.11** | **3.51** | **3.48** | **3.10** | **3.26** | **3.76** | **3.22** | **3.35** | **2.72** | **-** | **1.99** | **1.96** | **1.81** | **1.65** | **1.55** | **1.42** | **1.44** | **1.56** | **1.54** | **1.64** |
| DB | 0.99 | 0.81 | 0.89 | 0.86 | 0.84 | 0.84 | 0.88 | 0.75 | 0.79 | 0.72 | **-** | 0.47 | 0.44 | 0.40 | 0.34 | 0.32 | 0.32 | 0.29 | 0.28 | 0.21 | 0.23 |
| DC | 1.46 | 1.50 | 1.72 | 1.67 | 1.53 | 1.77 | 2.23 | 1.96 | 2.08 | 1.62 | **-** | 1.16 | 1.13 | 1.05 | 0.98 | 1.00 | 0.92 | 0.96 | 1.09 | 1.16 | 1.16 |
| DD | 0.85 | 0.79 | 0.90 | 0.94 | 0.73 | 0.64 | 0.65 | 0.51 | 0.48 | 0.38 | **-** | 0.36 | 0.38 | 0.37 | 0.33 | 0.23 | 0.19 | 0.18 | 0.19 | 0.17 | 0.25 |
| DE | **-** | **-** | **-** | **-** | **-** | **-** | **-** | **-** | **-** | **-** | **-** | **-** | **-** | **-** | **-** | **-** | **-** | **-** | **-** | **-** | **-** |
| **Romania^a^** | **-** | **-** | **-** | **-** | **-** | **-** | **-** | **-** | **-** | **-** | **-** | **-** | ***2.47*** | **-** | ***4.04*** | ***4.34*** | ***4.79*** | ***5.30*** | ***5.23*** | ***4.90*** | ***4.90*** |
| DB | **-** | **-** | **-** | **-** | **-** | **-** | **-** | **-** | **-** | **-** | **-** | **-** | *0.28* | **-** | *0.52* | *0.42* | *0.35* | *0.34* | *0.35* | *0.24* | *0.21* |
| DC | **-** | **-** | **-** | **-** | **-** | **-** | **-** | **-** | **-** | **-** | **-** | **-** | *2.09* | **-** | *2.73* | *2.99* | *3.46* | *3.91* | *3.62* | *3.45* | *3.41* |
| DD | **-** | **-** | **-** | **-** | **-** | **-** | **-** | **-** | **-** | **-** | **-** | **-** | *0.10* | **-** | *0.79* | *0.93* | *0.99* | *1.05* | *1.27* | *1.21* | *1.27* |
| DE | **-** | **-** | **-** | **-** | **-** | **-** | **-** | **-** | **-** | **-** | **-** | **-** | *<0.01* | **-** | *<0.01* | *<0.01* | ***-*** | ***-*** | *<0.01* | *<0.01* | *<0.01* |
| **Slovakia** | **-** | **-** | **1.95** | **2.49** | **2.73** | **2.47** | **2.48** | **2.16** | **3.40** | **3.04** | **3.86** | **3.92** | **4.12** | **-** | ***3.86*** | **3.49** | **4.51** | **4.36** | **4.71** | **4.62** | **-** |
| DB | **-** | **-** | 1.24 | 1.13 | 1.14 | 0.79 | 0.66 | 0.43 | 0.37 | 0.29 | 0.29 | 0.26 | 0.23 | **-** | *0.26* | 0.16 | 0.20 | 0.21 | 0.24 | 0.22 | **-** |
| DC | **-** | **-** | 0.67 | 1.24 | 1.44 | 1.56 | 1.70 | 1.63 | 2.92 | 2.42 | 3.08 | 3.12 | 3.35 | **-** | *3.10* | 2.80 | 3.54 | 3.42 | 3.71 | 3.64 | **-** |
| DD | **-** | **-** | 0.05 | 0.13 | 0.16 | 0.13 | 0.12 | 0.10 | 0.11 | 0.34 | 0.49 | 0.53 | 0.54 | **-** | *0.51* | 0.53 | 0.77 | 0.74 | 0.76 | 0.75 | **-** |
| DE | **-** | **-** | <0.01 | <0.01 | <0.01 | <0.01 | <0.01 | **-** | <0.01 | **-** | **-** | **-** | **-** | **-** | *<0.01* | **-** | **-** | **-** | **-** | **-** | **-** |
| **Slovenia** | **0.91** | **0.73** | **0.65** | **0.60** | **0.52** | **0.68** | **0.71** | **0.72** | **0.70** | **0.52** | **0.54** | **0.44** | **0.42** | **0.40** | **0.33** | **0.30** | **0.30** | **0.28** | **0.31** | **0.33** | **0.37** |
| DB | 0.28 | 0.19 | 0.12 | 0.11 | 0.10 | 0.09 | 0.09 | 0.07 | 0.06 | 0.06 | 0.03 | <0.01 | <0.01 | 0.01 | 0.01 | <0.01 | **-** | **-** | 0.01 | 0.03 | 0.04 |
| DC | 0.53 | 0.48 | 0.47 | 0.43 | 0.34 | 0.50 | 0.53 | 0.58 | 0.61 | 0.40 | 0.41 | 0.31 | 0.30 | 0.29 | 0.24 | 0.22 | 0.23 | 0.22 | 0.24 | 0.24 | 0.27 |
| DD | 0.10 | 0.06 | 0.05 | 0.06 | 0.08 | 0.09 | 0.09 | 0.06 | 0.04 | 0.06 | 0.10 | 0.12 | 0.11 | 0.10 | 0.08 | 0.08 | 0.07 | 0.06 | 0.06 | 0.06 | 0.06 |
| DE | **-** | **-** | **-** | **-** | **-** | **-** | **-** | **-** | **-** | **-** | **-** | **-** | **-** | **-** | **-** | **-** | **-** | **-** | **-** | **-** | **-** |
| Country, community consumption of cephalosporins (J01DB, J01DC, J01DD and J01DE combined); DB, first-generation cephalosporins (J01DB); DC, second-generation cephalosporins (J01DC); DD, third-generation cephalosporins (J01DD); DE, fourth-generation cephalosporins (J01DE); **-**, no consumption reported; Numbers reported in *italic* are total care data, i.e. community and hospital sector combined; ^a^Data for Romania have a coverage in 2009 limited to 30-40%; ^b^Data for Spain include private prescriptions as of 2016. | | | | | | | | | | | | | | | | | | | | | |
| **Spain^b^** | **2.57** | **2.62** | **2.54** | **2.33** | **2.06** | **1.98** | **2.04** | **1.81** | **1.83** | **1.67** | **1.79** | **1.65** | **1.56** | **1.56** | **1.53** | **1.44** | **1.57** | **1.64** | **1.64** | **2.24** | **2.27** |
| DB | 0.08 | 0.07 | 0.06 | 0.05 | 0.04 | 0.03 | 0.03 | 0.02 | 0.02 | 0.02 | 0.01 | 0.01 | 0.01 | 0.01 | 0.01 | 0.01 | 0.01 | 0.01 | 0.01 | 0.07 | 0.06 |
| DC | 1.82 | 1.93 | 1.88 | 1.70 | 1.48 | 1.40 | 1.46 | 1.31 | 1.27 | 1.13 | 1.19 | 1.08 | 1.00 | 1.02 | 1.04 | 1.00 | 1.13 | 1.25 | 1.24 | 1.65 | 1.68 |
| DD | 0.66 | 0.62 | 0.60 | 0.58 | 0.55 | 0.55 | 0.55 | 0.48 | 0.54 | 0.53 | 0.59 | 0.56 | 0.54 | 0.52 | 0.48 | 0.43 | 0.42 | 0.38 | 0.39 | 0.52 | 0.53 |
| DE | **-** | **-** | **-** | **-** | **-** | **-** | **-** | **-** | **-** | **-** | **-** | **-** | **-** | **-** | **-** | **-** | **-** | **-** | **-** | **-** | **-** |
| **Sweden** | **0.61** | **0.62** | **0.58** | **0.54** | **0.51** | **0.47** | **0.44** | **0.40** | **0.38** | **0.37** | **0.34** | **0.30** | **0.24** | **0.20** | **0.18** | **0.18** | **0.16** | **0.14** | **0.14** | **0.14** | **0.08** |
| DB | 0.40 | 0.41 | 0.40 | 0.38 | 0.37 | 0.35 | 0.33 | 0.30 | 0.28 | 0.28 | 0.26 | 0.23 | 0.18 | 0.14 | 0.15 | 0.15 | 0.13 | 0.12 | 0.11 | 0.11 | 0.08 |
| DC | 0.18 | 0.18 | 0.16 | 0.14 | 0.12 | 0.10 | 0.09 | 0.08 | 0.08 | 0.07 | 0.06 | 0.05 | 0.03 | 0.03 | <0.01 | **-** | **-** | **-** | **-** | **-** | **-** |
| DD | 0.04 | 0.03 | 0.03 | 0.02 | 0.02 | 0.02 | 0.02 | 0.02 | 0.02 | 0.02 | 0.02 | 0.03 | 0.03 | 0.03 | 0.03 | 0.03 | 0.03 | 0.03 | 0.03 | 0.03 | <0.01 |
| DE | **-** | **-** | **-** | **-** | **-** | **-** | **-** | **-** | **-** | **-** | **-** | **-** | **-** | **-** | **-** | **-** | **-** | **-** | **-** | **-** | **-** |
| **United Kingdom** | **1.08** | **0.96** | **0.81** | **0.76** | **0.78** | **0.77** | **0.77** | **0.76** | **0.78** | **0.79** | **0.80** | **0.71** | **0.58** | **0.55** | **0.42** | **0.35** | **0.34** | **0.32** | **0.27** | **0.24** | **0.23** |
| DB | 0.33 | 0.22 | 0.22 | 0.21 | 0.20 | 0.20 | 0.19 | 0.19 | 0.61 | 0.63 | 0.65 | 0.59 | 0.50 | 0.44 | 0.37 | 0.32 | 0.30 | 0.28 | 0.25 | 0.21 | 0.20 |
| DC | 0.71 | 0.71 | 0.58 | 0.54 | 0.57 | 0.56 | 0.57 | 0.56 | 0.17 | 0.16 | 0.14 | 0.12 | 0.08 | 0.10 | 0.04 | 0.03 | 0.03 | 0.02 | 0.02 | 0.01 | 0.01 |
| DD | 0.04 | 0.02 | 0.02 | 0.01 | 0.01 | 0.01 | 0.01 | 0.01 | 0.01 | 0.01 | 0.01 | <0.01 | <0.01 | <0.01 | <0.01 | <0.01 | 0.01 | 0.01 | 0.01 | 0.01 | 0.01 |
| DE | **-** | **-** | **-** | **-** | **-** | **-** | **-** | **-** | **-** | **-** | **-** | **-** | **-** | **-** | **-** | **-** | **-** | **-** | **-** | **-** | **-** |

Country, community consumption of cephalosporins (J01DB, J01DC, J01DD and J01DE combined); DB, first-generation cephalosporins (J01DB); DC, second-generation cephalosporins (J01DC); DD, third-generation cephalosporins (J01DD); DE, fourth-generation cephalosporins (J01DE); **-**, no consumption reported; Numbers reported in *italic* are total care data, i.e. community and hospital sector combined; ^a^Data for Romania have a coverage in 2009 limited to 30-40%; ^b^Data for Spain include private prescriptions as of 2016.

**Table S2. Consumption of cephalosporins (ATC J01DB, J01DC, J01DD and J01DE) in the community, expressed in packages per 1000 inhabitants per day, 23 EU/EEA countries, 2006-2017.**

| **Country** | **2006** | **2007** | **2008** | **2009** | **2010** | **2011** | **2012** | **2013** | **2014** | **2015** | **2016** | **2017** |  |
| --- | --- | --- | --- | --- | --- | --- | --- | --- | --- | --- | --- | --- | --- |
| **Austria** | **-** | **0.29** | **0.29** | **0.31** | **0.29** | **0.28** | **0.26** | **0.31** | **0.23** | **0.22** | **0.21** | **0.23** |  |
| DB | **-** | 0.05 | 0.06 | 0.06 | 0.06 | 0.06 | 0.05 | 0.06 | 0.06 | 0.06 | 0.06 | 0.06 |  |
| DC | **-** | 0.09 | 0.09 | 0.10 | 0.10 | 0.10 | 0.10 | 0.13 | 0.12 | 0.12 | 0.12 | 0.13 |  |
| DD | **-** | 0.15 | 0.15 | 0.15 | 0.14 | 0.13 | 0.11 | 0.12 | 0.05 | 0.04 | 0.03 | 0.04 |  |
| DE | **-** | **-** | **-** | **-** | **-** | **-** | **-** | **-** | **-** | **-** | **-** | **-** |  |
| **Belgium^a^** | **-** | **0.19** | **0.16** | **0.14** | **0.13** | **0.12** | **0.12** | **0.12** | **0.11** | **0.11** | **0.12** | **0.11** |  |
| DB | **-** | 0.05 | 0.05 | 0.05 | 0.04 | 0.04 | 0.04 | 0.04 | 0.03 | 0.03 | 0.03 | 0.02 |  |
| DC | **-** | 0.14 | 0.11 | 0.10 | 0.09 | 0.08 | 0.08 | 0.08 | 0.07 | 0.08 | 0.10 | 0.09 |  |
| DD | **-** | <0.01 | <0.01 | <0.01 | <0.01 | <0.01 | <0.01 | <0.01 | <0.01 | <0.01 | <0.01 | <0.01 |  |
| DE | **-** | **-** | **-** | **-** | **-** | **-** | **-** | **-** | **-** | **-** | **-** | **-** |  |
| **Bulgaria** | **0.45** | **0.48** | **0.51** | **0.51** | **0.52** | **0.55** | **0.51** | **0.57** | **0.64** | **0.68** | **0.65** | **0.66** |  |
| DB | 0.33 | 0.32 | 0.28 | 0.21 | 0.19 | 0.18 | 0.13 | 0.12 | 0.10 | 0.07 | 0.05 | 0.04 |  |
| DC | 0.10 | 0.13 | 0.17 | 0.21 | 0.22 | 0.22 | 0.21 | 0.24 | 0.29 | 0.37 | 0.35 | 0.38 |  |
| DD | 0.02 | 0.03 | 0.06 | 0.08 | 0.11 | 0.15 | 0.17 | 0.21 | 0.25 | 0.24 | 0.25 | 0.24 |  |
| DE | <0.01 | <0.01 | <0.01 | <0.01 | <0.01 | <0.01 | <0.01 | <0.01 | <0.01 | <0.01 | <0.01 | <0.01 |  |
| **Croatia** | **-** | **0.71** | **0.70** | **0.63** | **0.54** | **0.44** | **0.50** | **0.44** | **0.42** | **0.39** | **0.35** | **0.36** |  |
| DB | **-** | 0.45 | 0.38 | 0.29 | 0.23 | 0.19 | 0.17 | 0.17 | 0.17 | 0.15 | 0.13 | 0.12 |  |
| DC | **-** | 0.15 | 0.22 | 0.22 | 0.19 | 0.15 | 0.22 | 0.20 | 0.21 | 0.20 | 0.19 | 0.18 |  |
| DD | **-** | 0.11 | 0.11 | 0.12 | 0.11 | 0.11 | 0.11 | 0.07 | 0.04 | 0.04 | 0.04 | 0.06 |  |
| DE | **-** | <0.01 | <0.01 | <0.01 | **-** | <0.01 | **-** | **-** | **-** | **-** | **-** | **-** |  |
| **Czech Republic** | **-** | **0.18** | **-** | **-** | **0.47** | **0.46** | **0.35** | **0.38** | **0.47** | **0.48** | **-** | **-** |  |
| DB | **-** | 0.04 | **-** | **-** | 0.04 | 0.04 | 0.03 | 0.03 | 0.03 | 0.03 | **-** | **-** |  |
| DC | **-** | 0.15 | **-** | **-** | 0.23 | 0.23 | 0.19 | 0.24 | 0.26 | 0.26 | **-** | **-** |  |
| DD | **-** | <0.01 | **-** | **-** | 0.19 | 0.19 | 0.13 | 0.12 | 0.17 | 0.19 | **-** | **-** |  |
| DE | **-** | **-** | **-** | **-** | 0.01 | 0.01 | 0.01 | <0.01 | 0.01 | 0.01 | **-** | **-** |  |
| **Denmark** | **-** | **<0.01** | **0.01** | **0.01** | **0.01** | **0.01** | **0.01** | **<0.01** | **<0.01** | **<0.01** | **<0.01** | **<0.01** |  |
| DB | **-** | <0.01 | <0.01 | <0.01 | <0.01 | <0.01 | <0.01 | <0.01 | <0.01 | <0.01 | <0.01 | <0.01 |  |
| DC | **-** | <0.01 | <0.01 | <0.01 | <0.01 | 0.01 | <0.01 | <0.01 | <0.01 | <0.01 | <0.01 | <0.01 |  |
| DD | **-** | <0.01 | <0.01 | <0.01 | <0.01 | <0.01 | <0.01 | <0.01 | <0.01 | <0.01 | <0.01 | <0.01 |  |
| DE | **-** | **-** | **-** | **-** | **-** | **-** | **-** | **-** | **-** | **-** | **-** | **-** |  |
| Country, community consumption of cephalosporins (J01DB, J01DC, J01DD and J01DE combined); DB, first-generation cephalosporins (J01DB); DC, second-generation cephalosporins (J01DC); DD, third-generation cephalosporins (J01DD); DE, fourth-generation cephalosporins (J01DE); **-**, no consumption reported; Numbers reported in *italic* are total care data, i.e. community and hospital sector combined; ^a^Data for Belgium are slightly overestimated as of 2016 (nursing homes counting units versus packages before 2016); ^b^Data for the Netherlands are based on average package size; ^c^Data for Spain include private prescriptions as of 2016. | | | | | | | | | | | | | |
| **Estonia** | **0.21** | **0.20** | **0.21** | **0.18** | **0.18** | **0.18** | **0.17** | **0.18** | **0.18** | **0.18** | **0.17** | **0.17** |  |
| DB | 0.10 | 0.08 | 0.08 | 0.07 | 0.07 | 0.07 | 0.07 | 0.07 | 0.07 | 0.06 | 0.05 | 0.06 |  |
| DC | 0.11 | 0.12 | 0.12 | 0.11 | 0.11 | 0.11 | 0.10 | 0.11 | 0.11 | 0.12 | 0.12 | 0.11 |  |
| DD | <0.01 | <0.01 | <0.01 | <0.01 | <0.01 | <0.01 | <0.01 | <0.01 | <0.01 | <0.01 | <0.01 | <0.01 |  |
| DE | **-** | **-** | <0.01 | **-** | **-** | <0.01 | <0.01 | **-** | **-** | **-** | **-** | **-** |  |
| **Finland** | **-** | **-** | **0.41** | **0.41** | **0.41** | **0.41** | **0.41** | **0.40** | **0.40** | **0.37** | **0.37** | **0.35** |  |
| DB | **-** | **-** | 0.39 | 0.39 | 0.40 | 0.40 | 0.39 | 0.39 | 0.39 | 0.37 | 0.36 | 0.35 |  |
| DC | **-** | **-** | 0.01 | 0.01 | 0.01 | 0.01 | 0.01 | 0.01 | 0.01 | 0.01 | 0.01 | <0.01 |  |
| DD | **-** | **-** | <0.01 | <0.01 | <0.01 | <0.01 | <0.01 | <0.01 | <0.01 | <0.01 | <0.01 | <0.01 |  |
| DE | **-** | **-** | **-** | **-** | **-** | **-** | **-** | **-** | **-** | **-** | **-** | **-** |  |
| **France** | **-** | **-** | **-** | **-** | **1.14** | **1.12** | **1.05** | **1.00** | **0.89** | **0.94** | **0.84** | **0.73** |  |
| DB | **-** | **-** | **-** | **-** | 0.03 | 0.02 | 0.02 | 0.02 | 0.02 | 0.01 | 0.01 | 0.01 |  |
| DC | **-** | **-** | **-** | **-** | 0.20 | 0.16 | 0.17 | 0.16 | 0.13 | 0.12 | 0.11 | 0.09 |  |
| DD | **-** | **-** | **-** | **-** | 0.91 | 0.94 | 0.86 | 0.83 | 0.75 | 0.80 | 0.73 | 0.63 |  |
| DE | **-** | **-** | **-** | **-** | <0.01 | <0.01 | <0.01 | <0.01 | <0.01 | <0.01 | <0.01 | <0.01 |  |
| **Greece** | ***2.52*** | ***2.63*** | ***2.48*** | **1.46** | ***2.12*** | **1.25** | **1.07** | **1.10** | **1.26** | **1.14** | **1.15** | **1.17** |  |
| DB | *0.04* | *0.04* | *0.03* | 0.02 | *0.01* | <0.01 | <0.01 | <0.01 | <0.01 | <0.01 | <0.01 | <0.01 |  |
| DC | *2.16* | *2.22* | *2.11* | 1.33 | *1.84* | 1.16 | 1.01 | 1.03 | 1.17 | 1.07 | 1.07 | 1.07 |  |
| DD | *0.31* | *0.34* | *0.31* | 0.11 | *0.25* | 0.08 | 0.06 | 0.06 | 0.09 | 0.07 | 0.07 | 0.09 |  |
| DE | *0.02* | *0.03* | *0.03* | <0.01 | *0.03* | 0.01 | <0.01 | <0.01 | <0.01 | <0.01 | <0.01 | 0.01 |  |
| **Iceland** | **-** | **-** | **-** | **-** | ***0.15*** | ***0.16*** | ***0.17*** | ***0.20*** | **0.10** | **0.10** | **0.11** | **0.12** |  |
| DB | **-** | **-** | **-** | **-** | *0.08* | *0.08* | *0.09* | *0.11* | 0.09 | 0.10 | 0.11 | 0.12 |  |
| DC | **-** | **-** | **-** | **-** | *0.05* | *0.05* | *0.05* | *0.04* | 0.01 | <0.01 | <0.01 | **-** |  |
| DD | **-** | **-** | **-** | **-** | *0.02* | *0.02* | *0.02* | *0.05* | **-** | <0.01 | <0.01 | <0.01 |  |
| DE | **-** | **-** | **-** | **-** | ***-*** | ***-*** | ***-*** | ***-*** | **-** | **-** | **-** | **-** |  |
| **Ireland** | **-** | **0.39** | **-** | **-** | **0.26** | **0.28** | **0.29** | **0.28** | **0.24** | **0.26** | **0.25** | **0.23** |  |
| DB | **-** | 0.06 | **-** | **-** | 0.04 | 0.04 | 0.05 | 0.05 | 0.05 | 0.06 | 0.07 | 0.08 |  |
| DC | **-** | 0.29 | **-** | **-** | 0.18 | 0.20 | 0.21 | 0.20 | 0.16 | 0.17 | 0.16 | 0.13 |  |
| DD | **-** | 0.05 | **-** | **-** | 0.04 | 0.04 | 0.04 | 0.03 | 0.03 | 0.02 | 0.02 | 0.02 |  |
| DE | **-** | **-** | **-** | **-** | **-** | **-** | **-** | **-** | **-** | **-** | **-** | **-** |  |
| Country, total national use of cephalosporins (J01DB, J01DC, J01DD and J01DE combined); DB, first-generation cephalosporins (J01DB); DC, second-generation cephalosporins (J01DC); DD, third-generation cephalosporins (J01DD); DE, fourth-generation cephalosporins (J01DE); **-**, no consumption reported; Numbers reported in *italic* are total care data, i.e. community and hospital sector combined; ^a^Data for Belgium are slightly overestimated as of 2016 (nursing homes counting units versus packages before 2016); ^b^Data for the Netherlands are based on average package size; ^c^Data for Spain include private prescriptions as of 2016. | | | | | | | | | | | | | |
| **Italy** | **-** | **-** | **1.59** | **-** | **1.49** | **1.46** | **1.37** | **1.41** | **1.33** | **1.30** | **1.22** | **1.05** |  |
| DB | **-** | **-** | 0.07 | **-** | 0.05 | 0.05 | 0.04 | 0.04 | 0.04 | 0.04 | 0.04 | 0.04 |  |
| DC | **-** | **-** | 0.19 | **-** | 0.13 | 0.12 | 0.10 | 0.09 | 0.08 | 0.06 | 0.05 | 0.04 |  |
| DD | **-** | **-** | 1.31 | **-** | 1.28 | 1.27 | 1.21 | 1.26 | 1.19 | 1.19 | 1.12 | 0.97 |  |
| DE | **-** | **-** | 0.02 | **-** | 0.02 | 0.02 | 0.02 | 0.02 | 0.02 | 0.02 | 0.02 | 0.01 |  |
| **Latvia** | **-** | **-** | **-** | **-** | **0.29** | **0.19** | **0.16** | **0.18** | **0.16** | **0.16** | **0.17** | **0.17** |  |
| DB | **-** | **-** | **-** | **-** | 0.16 | 0.11 | 0.07 | 0.08 | 0.06 | 0.07 | 0.06 | 0.06 |  |
| DC | **-** | **-** | **-** | **-** | 0.03 | 0.03 | 0.03 | 0.04 | 0.04 | 0.04 | 0.04 | 0.05 |  |
| DD | **-** | **-** | **-** | **-** | 0.11 | 0.06 | 0.06 | 0.07 | 0.06 | 0.05 | 0.06 | 0.06 |  |
| DE | **-** | **-** | **-** | **-** | **-** | **-** | **-** | **-** | **-** | **-** | **-** | **-** |  |
| **Lithuania** | **-** | ***0.72*** | ***1.10*** | ***0.66*** | ***0.72*** | ***0.78*** | **0.20** | **0.22** | **0.19** | **0.19** | **0.19** | **0.19** |  |
| DB | **-** | *0.44* | *0.58* | *0.28* | *0.32* | *0.29* | 0.08 | 0.10 | 0.09 | 0.09 | 0.09 | 0.09 |  |
| DC | **-** | *0.16* | *0.33* | *0.27* | *0.29* | *0.36* | 0.10 | 0.11 | 0.09 | 0.09 | 0.10 | 0.10 |  |
| DD | **-** | *0.12* | *0.18* | *0.10* | *0.11* | *0.12* | 0.01 | 0.01 | 0.01 | 0.01 | 0.01 | 0.01 |  |
| DE | **-** | *<0.01* | *<0.01* | *<0.01* | *<0.01* | *<0.01* | <0.01 | **-** | <0.01 | <0.01 | **-** | **-** |  |
| **Luxembourg** | **0.45** | **0.41** | **0.35** | **0.37** | **0.35** | **0.32** | **0.31** | **0.32** | **0.29** | **0.30** | **0.27** | **-** |  |
| DB | 0.05 | 0.04 | 0.04 | 0.04 | 0.04 | 0.03 | 0.03 | 0.03 | 0.03 | 0.03 | 0.02 | **-** |  |
| DC | 0.38 | 0.35 | 0.29 | 0.31 | 0.29 | 0.26 | 0.25 | 0.26 | 0.23 | 0.24 | 0.21 | **-** |  |
| DD | 0.02 | 0.02 | 0.02 | 0.02 | 0.02 | 0.03 | 0.03 | 0.03 | 0.03 | 0.03 | 0.03 | **-** |  |
| DE | <0.01 | **-** | **-** | **-** | **-** | **-** | <0.01 | **-** | <0.01 | **-** | <0.01 | **-** |  |
| **Netherlands^b^** | **-** | **-** | **0.01** | **0.01** | **-** | **-** | **-** | **-** | **-** | **-** | **-** | **-** |  |
| DB | **-** | **-** | <0.01 | <0.01 | **-** | **-** | **-** | **-** | **-** | **-** | **-** | **-** |  |
| DC | **-** | **-** | <0.01 | <0.01 | **-** | **-** | **-** | **-** | **-** | **-** | **-** | **-** |  |
| DD | **-** | **-** | <0.01 | <0.01 | **-** | **-** | **-** | **-** | **-** | **-** | **-** | **-** |  |
| DE | **-** | **-** | **-** | **-** | **-** | **-** | **-** | **-** | **-** | **-** | **-** | **-** |  |
| **Portugal** | **-** | **-** | **0.30** | **0.29** | **0.26** | **0.22** | **0.20** | **0.18** | **0.18** | **0.19** | **0.15** | **0.14** |  |
| DB | **-** | **-** | 0.07 | 0.06 | 0.06 | 0.05 | 0.04 | 0.04 | 0.04 | 0.04 | 0.03 | 0.02 |  |
| DC | **-** | **-** | 0.11 | 0.10 | 0.09 | 0.08 | 0.08 | 0.07 | 0.07 | 0.08 | 0.08 | 0.08 |  |
| DD | **-** | **-** | 0.12 | 0.12 | 0.11 | 0.10 | 0.08 | 0.07 | 0.07 | 0.08 | 0.04 | 0.04 |  |
| DE | **-** | **-** | **-** | **-** | **-** | **-** | **-** | **-** | **-** | **-** | **-** | **-** |  |
| Country, total national use of cephalosporins (J01DB, J01DC, J01DD and J01DE combined); DB, first-generation cephalosporins (J01DB); DC, second-generation cephalosporins (J01DC); DD, third-generation cephalosporins (J01DD); DE, fourth-generation cephalosporins (J01DE); **-**, no consumption reported; Numbers reported in *italic* are total care data, i.e. community and hospital secor combined; ^a^Data for Belgium are slightly overestimated as of 2016 (nursing homes counting units versus packages before 2016); ^b^Data for the Netherlands are based on average package size; ^c^Data for Spain include private prescriptions as of 2016. | | | | | | | | | | | | | |
| **Slovakia** | **-** | **-** | **-** | **-** | **-** | ***0.70*** | **0.52** | **0.68** | **0.65** | **0.69** | **0.69** | **-** |  |
| DB | **-** | **-** | **-** | **-** | **-** | *0.07* | 0.05 | 0.06 | 0.06 | 0.07 | 0.07 | **-** |  |
| DC | **-** | **-** | **-** | **-** | **-** | *0.39* | 0.35 | 0.44 | 0.44 | 0.48 | 0.48 | **-** |  |
| DD | **-** | **-** | **-** | **-** | **-** | *0.24* | 0.12 | 0.18 | 0.15 | 0.14 | 0.14 | **-** |  |
| DE | **-** | **-** | **-** | **-** | **-** | *<0.01* | <0.01 | <0.01 | **-** | **-** | **-** | **-** |  |
| **Slovenia** | **-** | **0.09** | **0.07** | **0.06** | **0.06** | **0.05** | **0.04** | **0.04** | **0.03** | **0.04** | **0.04** | **0.05** |  |
| DB | **-** | 0.01 | <0.01 | <0.01 | <0.01 | <0.01 | **-** | **-** | **-** | <0.01 | 0.01 | 0.01 |  |
| DC | **-** | 0.07 | 0.05 | 0.04 | 0.04 | 0.03 | 0.03 | 0.03 | 0.03 | 0.03 | 0.03 | 0.03 |  |
| DD | **-** | 0.02 | 0.02 | 0.01 | 0.01 | 0.01 | 0.01 | 0.01 | 0.01 | 0.01 | 0.01 | 0.01 |  |
| DE | **-** | **-** | **-** | **-** | **-** | **-** | **-** | **-** | **-** | **-** | **-** | **-** |  |
| **Spain^c^** | **-** | **-** | **-** | **-** | **0.21** | **0.20** | **0.19** | **0.18** | **0.16** | **0.15** | **0.21** | **0.21** |  |
| DB | **-** | **-** | **-** | **-** | 0.01 | 0.01 | <0.01 | <0.01 | <0.01 | <0.01 | 0.01 | 0.01 |  |
| DC | **-** | **-** | **-** | **-** | 0.13 | 0.13 | 0.12 | 0.12 | 0.11 | 0.11 | 0.13 | 0.13 |  |
| DD | **-** | **-** | **-** | **-** | 0.07 | 0.07 | 0.06 | 0.06 | 0.04 | 0.04 | 0.07 | 0.07 |  |
| DE | **-** | **-** | **-** | **-** | <0.01 | <0.01 | <0.01 | **-** | **-** | **-** | **-** | **-** |  |
| **Sweden** | **-** | **-** | **-** | **0.05** | **0.04** | **0.04** | **0.04** | **0.03** | **0.03** | **0.03** | **0.03** | **0.02** |  |
| DB | **-** | **-** | **-** | 0.04 | 0.04 | 0.03 | 0.03 | 0.03 | 0.03 | 0.02 | 0.02 | 0.02 |  |
| DC | **-** | **-** | **-** | <0.01 | <0.01 | <0.01 | **-** | **-** | **-** | **-** | **-** | **-** |  |
| DD | **-** | **-** | **-** | <0.01 | 0.01 | 0.01 | 0.01 | 0.01 | <0.01 | 0.01 | 0.01 | <0.01 |  |
| DE | **-** | **-** | **-** | **-** | **-** | **-** | **-** | **-** | **-** | **-** | **-** | **-** |  |
| **United Kingdom** | **-** | **-** | **-** | **-** | **-** | **-** | **-** | **-** | **-** | **<0.01** | **-** | **-** |  |
| DB | **-** | **-** | **-** | **-** | **-** | **-** | **-** | **-** | **-** | <0.01 | **-** | **-** |  |
| DC | **-** | **-** | **-** | **-** | **-** | **-** | **-** | **-** | **-** | <0.01 | **-** | **-** |  |
| DD | **-** | **-** | **-** | **-** | **-** | **-** | **-** | **-** | **-** | **-** | **-** | **-** |  |
| DE | **-** | **-** | **-** | **-** | **-** | **-** | **-** | **-** | **-** | **-** | **-** | **-** |  |

Country, total national use of cephalosporins (J01DB, J01DC, J01DD and J01DE combined); DB, first-generation cephalosporins (J01DB); DC, second-generation cephalosporins (J01DC); DD, third-generation cephalosporins (J01DD); DE, fourth-generation cephalosporins (J01DE); **-**, no consumption reported; Numbers reported in *italic* are total care data, i.e. community and hospital sector combined; ^a^Data for Belgium are slightly overestimated as of 2016 (nursing homes counting units versus packages before 2016); ^b^Data for the Netherlands are based on average package size; ^c^Data for Spain include private prescriptions as of 2016.

**
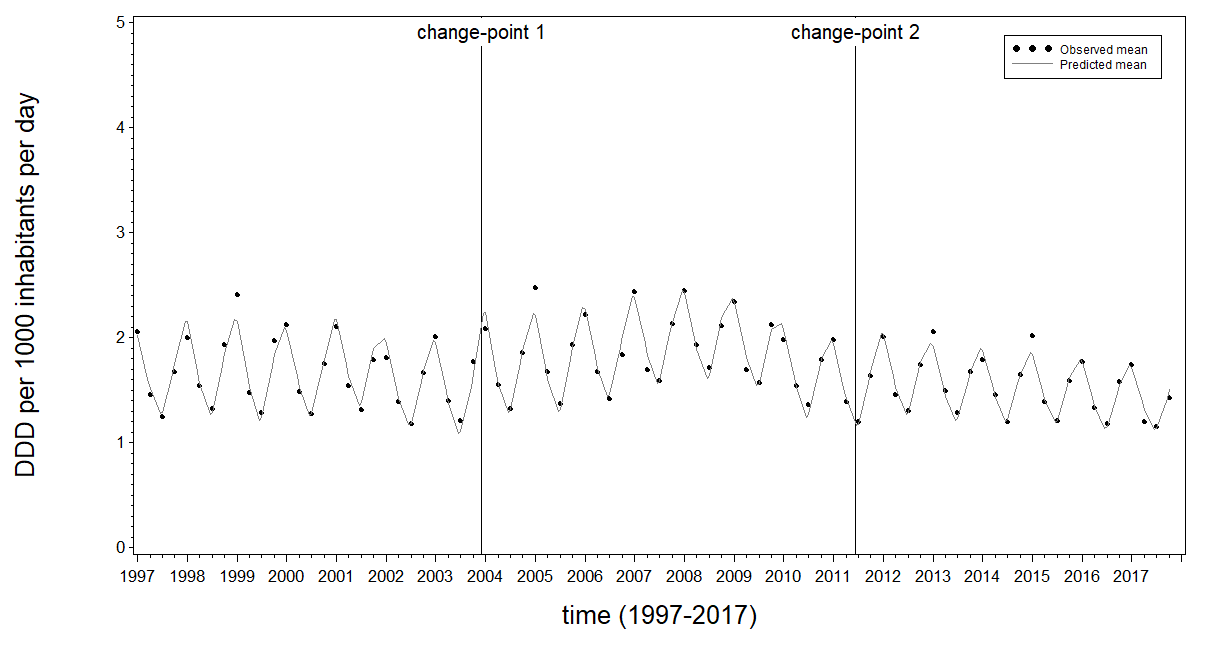
**

**Figure S1. Average of observed (dots) and predicted (solid line) consumption of cephalosporins (ATC J01DB, J01DC, J01DD and J01DE) in the community expressed in DDD (ATC/DDD index 2019) per 1000 inhabitants per day and based on quarterly data, 25 EU/EEA countries, 1997-2017.**

**
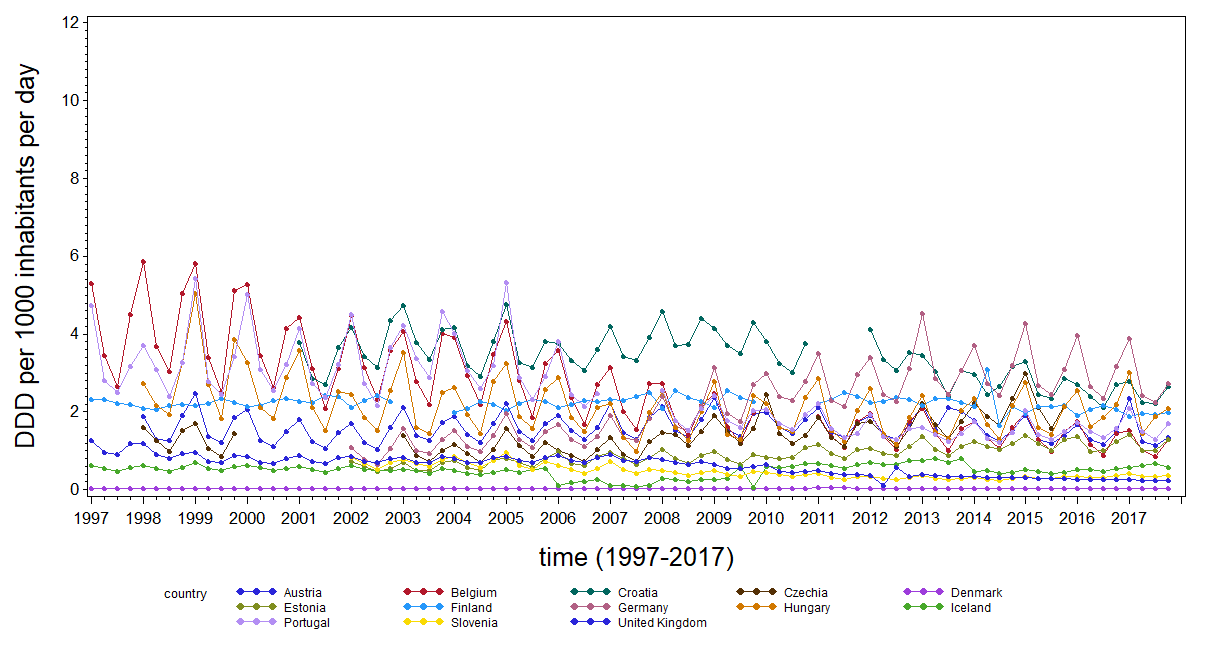
**

**Figure S2. Seasonal variation in consumption of cephalosporins (ATC J01DB, J01DC, J01DD and J01DE) in the community, expressed in DDD (ATC/DDD index 2019) per 1000 inhabitants per day, 13 EU/EEA countries reporting consumption per quarter for at least 15 years, 1997-2017.**


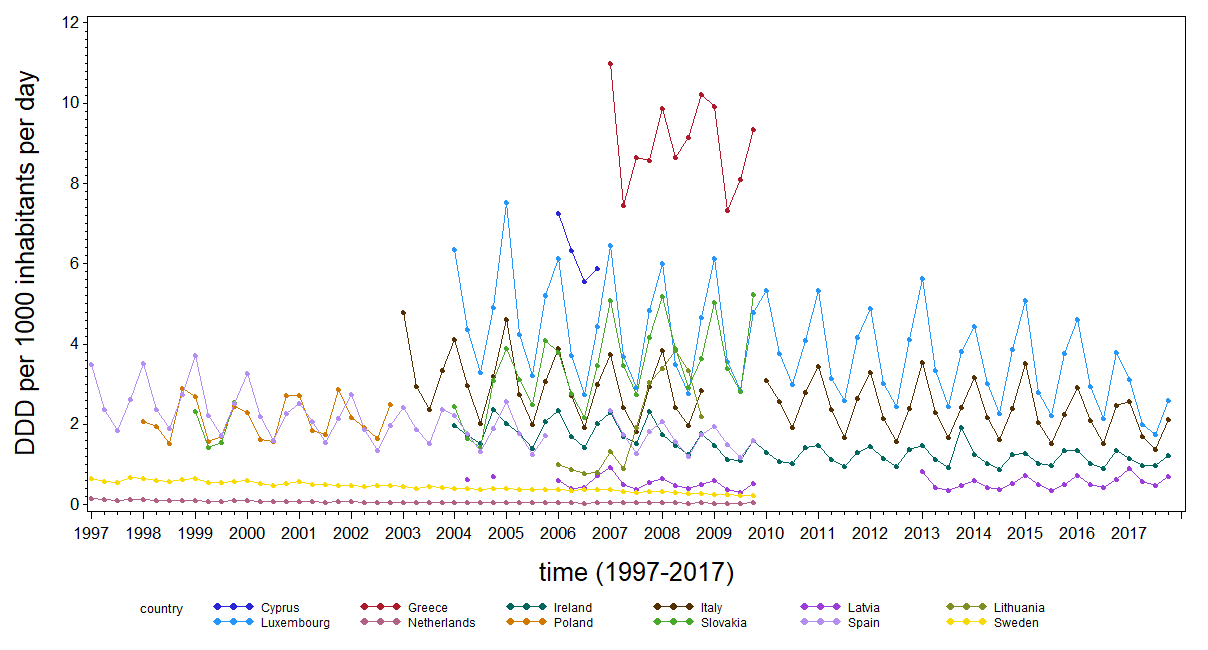


**Figure S3. Seasonal variation in consumption of cephalosporins (ATC J01DB, J01DC, J01DD and J01DE) in the community, expressed in DDD (ATC/DDD index 2019) per 1000 inhabitants per day, 12 EU countries reporting consumption per quarter for less than 15 years, 1997-2017. For Cyprus, total care data are used. For Spain, private prescriptions are included as of 2016.**

^
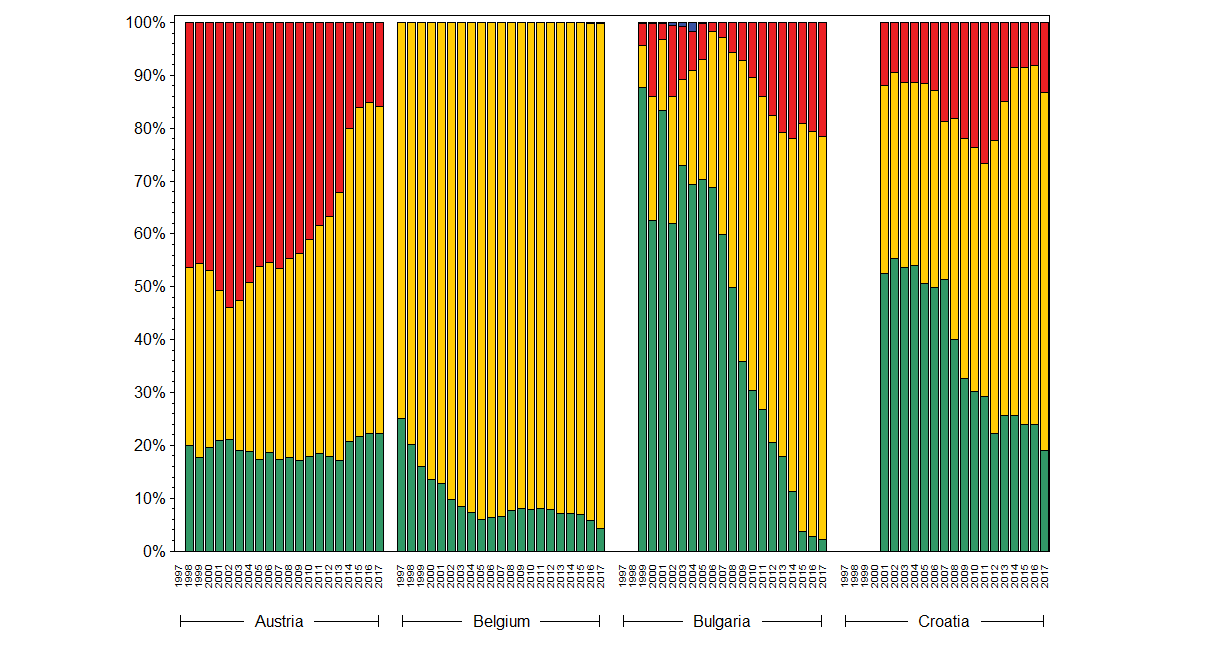
^

^
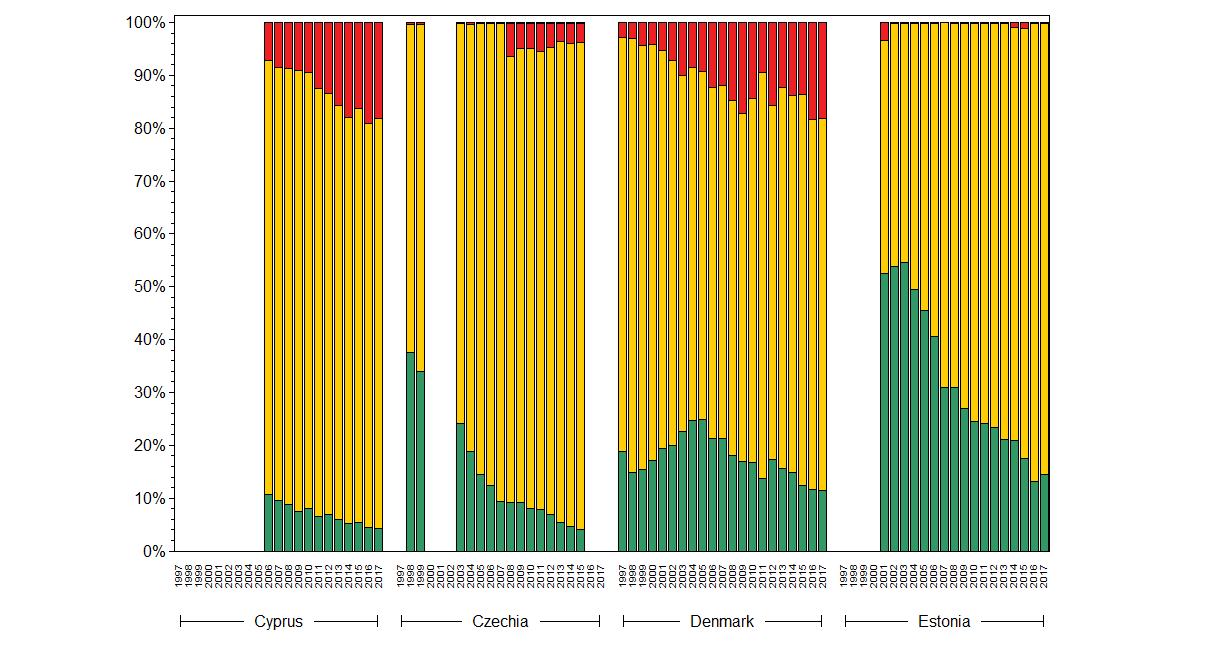
^

= first-generation cephalosporins (J01DB), = second-generation cephalosporins (J01DC),
 = third-generation cephalosporins (J01DD), = fourth-generation cephalosporins (J01DE)

**Figure S4. Composition of cephalosporin (ATC J01DB, J01DC, J01DD and J01DE) consumption in the community, expressed in DDD (ATC/DDD index 2019) per 1000 inhabitants per day, 30 EU/EEA countries, 1997-2017. For Cyprus and Romania, total care data, i.e. community and hospital sector combined, are used. For Spain, private prescription are included as of 2016. For Romania, data have a coverage in 2009 limited to 30-40%.**

^
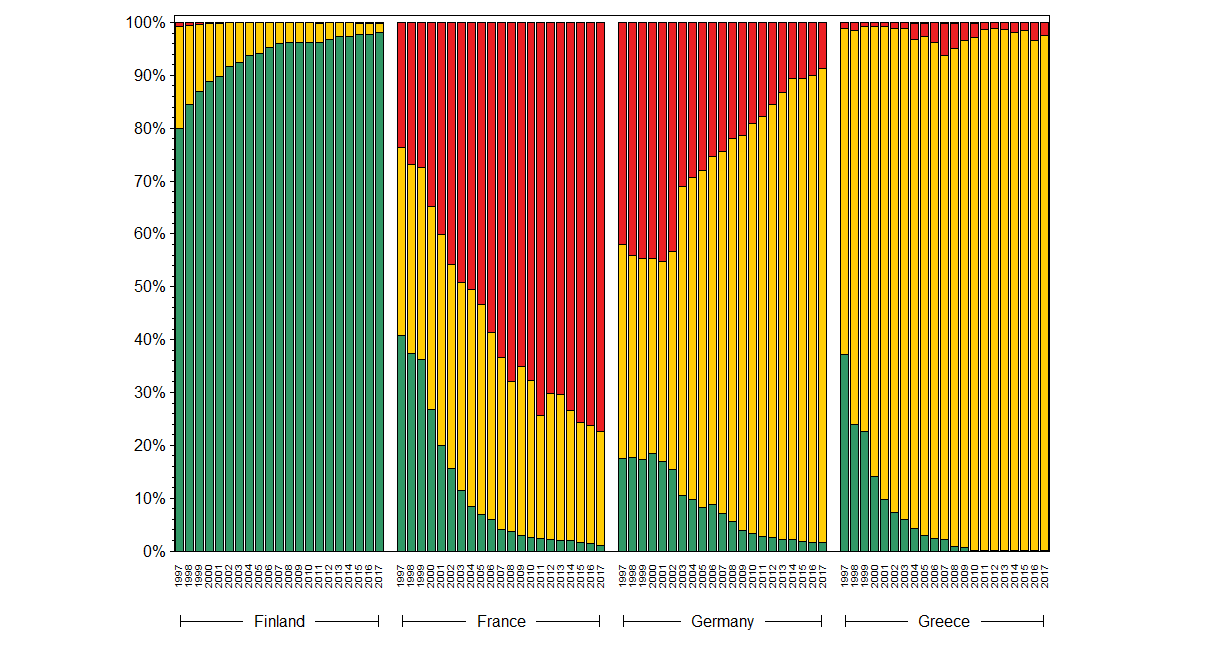
^

^
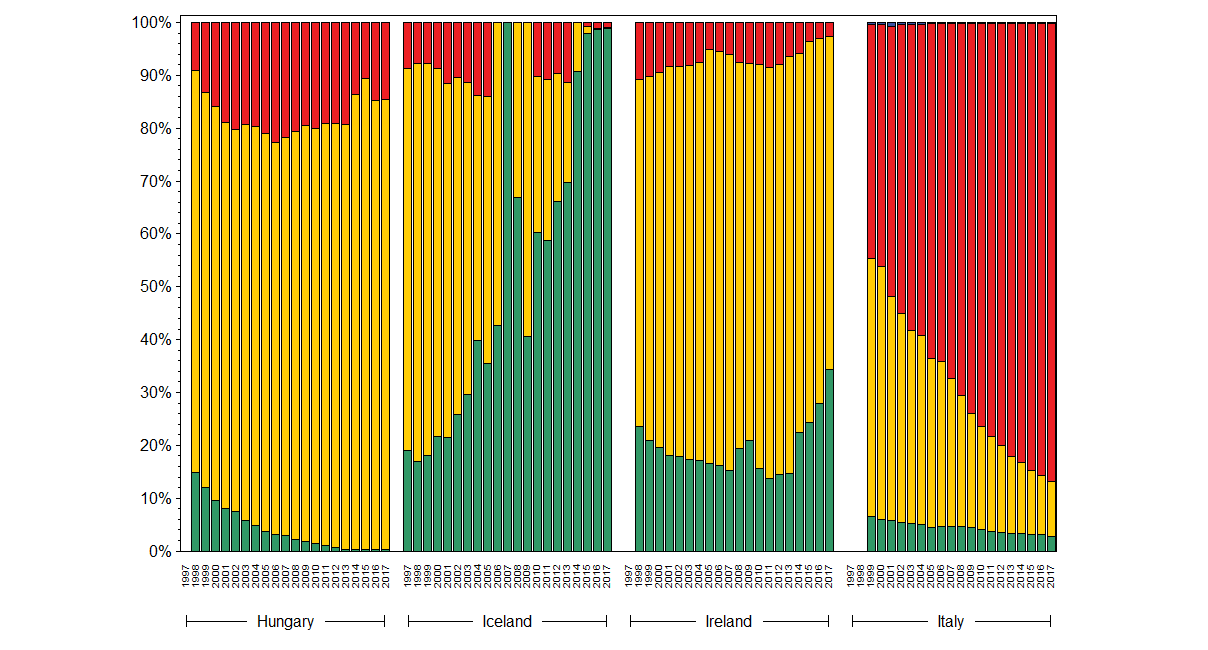
^

= first-generation cephalosporins (J01DB), = second-generation cephalosporins (J01DC),
 = third-generation cephalosporins (J01DD), = fourth-generation cephalosporins (J01DE)

**Figure S4.** *Continued*

^
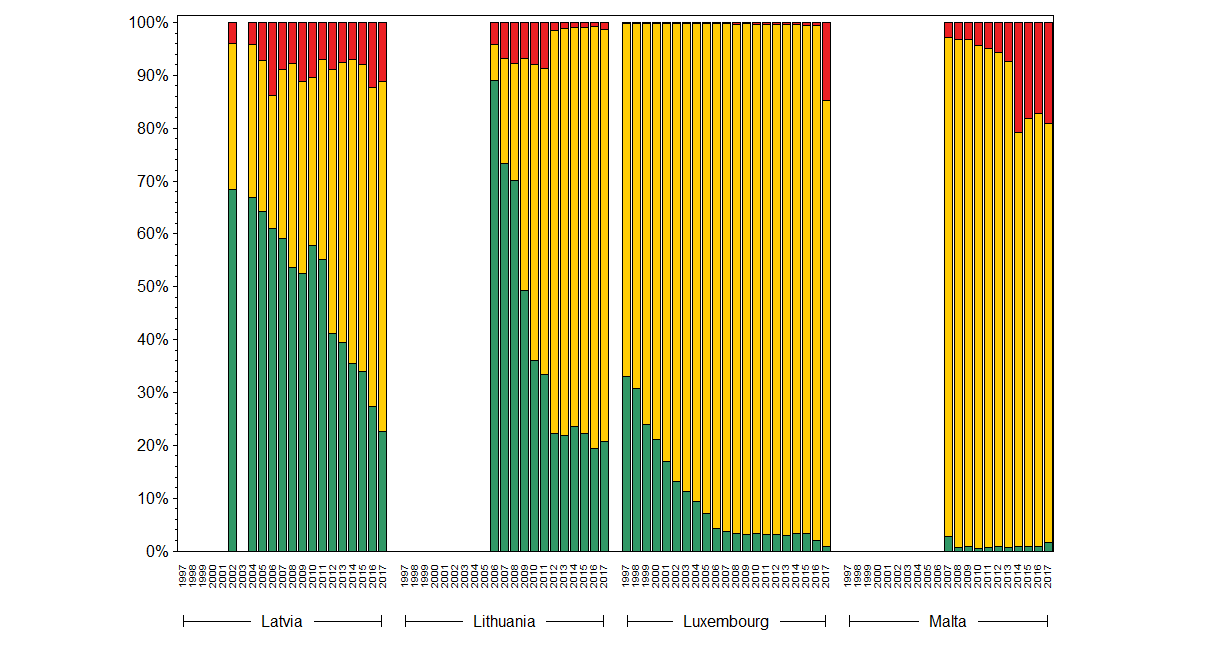
^

^
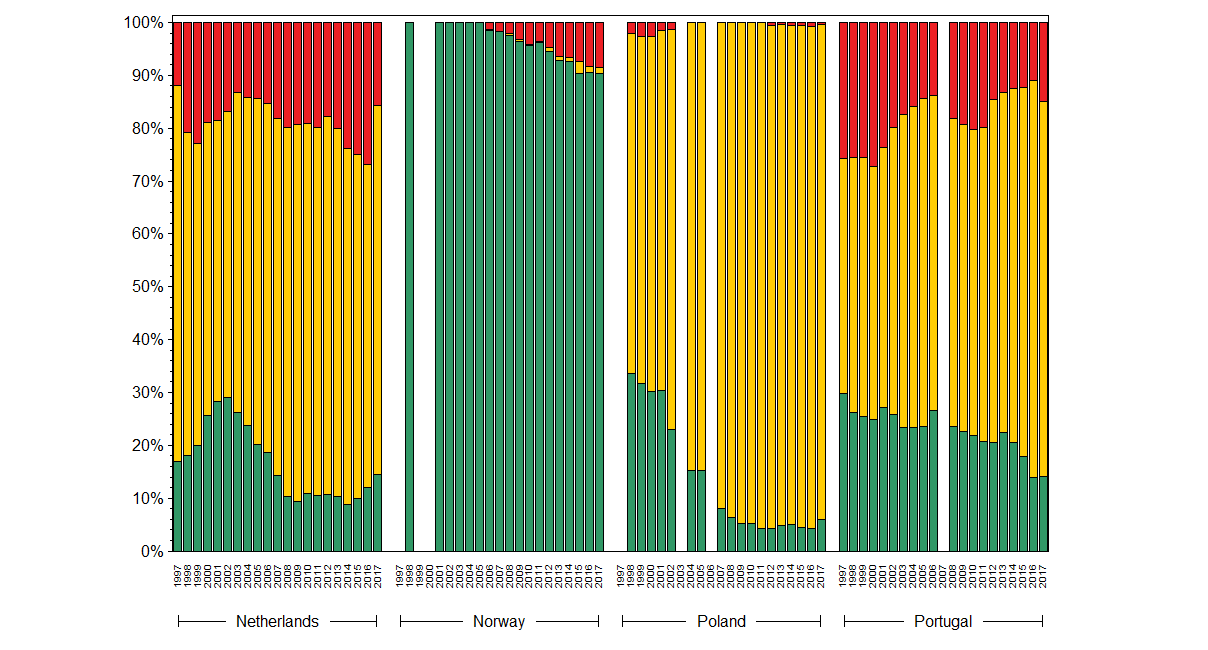
^

= first-generation cephalosporins (J01DB), = second-generation cephalosporins (J01DC),
 = third-generation cephalosporins (J01DD), = fourth-generation cephalosporins (J01DE)

**Figure S4.** *Continued*

^
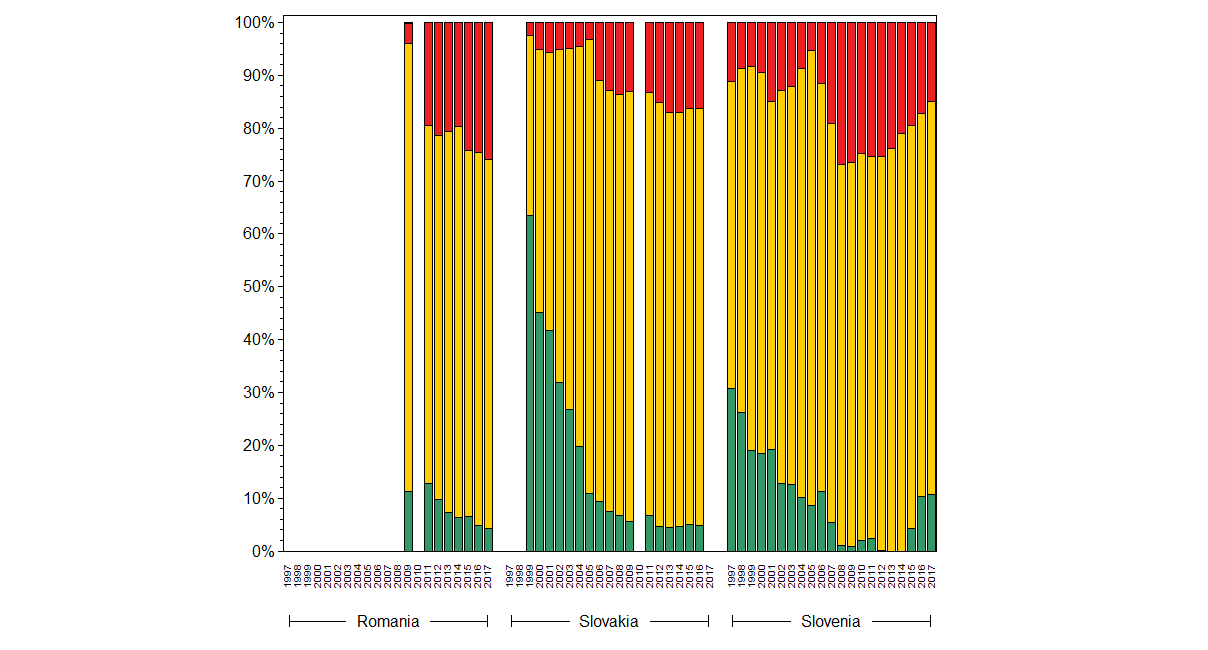
^

^
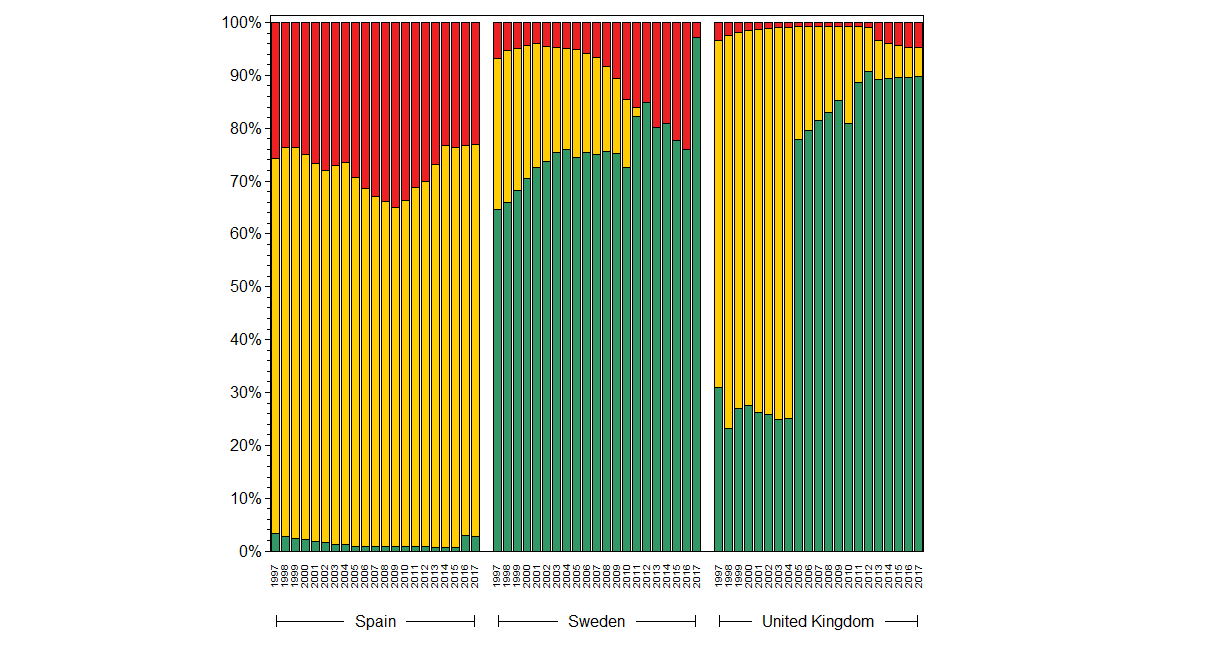
^

= first-generation cephalosporins (J01DB), = second-generation cephalosporins (J01DC),
 = third-generation cephalosporins (J01DD), = fourth-generation cephalosporins (J01DE)

**Figure S4.** *Continued*
